# ONC201 (Dordaviprone) Induces Integrated Stress Response and Death in Cervical Cancer Cells

**DOI:** 10.3390/biom15040463

**Published:** 2025-03-21

**Authors:** Sneha O. Pathak, Sonal M. Manohar

**Affiliations:** Department of Biological Sciences, Sunandan Divatia of School of Science, SVKM’s NMIMS (Deemed-to-be) University, Vile Parle (West), Mumbai 400056, India

**Keywords:** ONC201, dordaviprone, TIC10, cervical cancer, integrated stress response, apoptosis, small molecule

## Abstract

Cervical cancer is a leading cause of death in women globally. Systemic chemotherapy offers only limited therapeutic benefit for advanced-stage disease due to toxicity and drug resistance. ONC201 (also known as TIC10 or dordaviprone) is a TRAIL (TNF-Related Apoptosis-Inducing Ligand) and cIpP (caseinolytic protease) agonist currently in Phase II clinical trials for different types of cancer. In the present study, we investigated the anticancer potential of ONC201 in HPV-positive cervical cancer cell lines. ONC201 exerted significant cytotoxicity and inhibited the clonogenic potential of cervical cancer cells. It induced integrated stress response along with S/G2-M arrest and apoptosis in both cell lines. Yet, surprisingly, well-known targets of ONC201 viz. TRAIL, DR5 (death receptor 5) and cIpP were found to be upregulated only in HeLa but not in SiHa cells in response to ONC201 treatment. In addition, expression of BNIP3 and Beclin-1 (both involved in regulation of autophagy) increased in response to certain doses of ONC201. Furthermore, ONC201 exhibited synergism in combination with standard drugs against cervical cancer cells. This study provides a proof of concept for the anticancer activity of versatile drug ONC201 against cervical cancer cells and also delineates its mechanism of action.

## 1. Introduction

A major public health concern worldwide, cervical cancer is highly common in low- and middle-income nations [1]. Cervical cancer (CC) is mainly averted with established effectiveness of preventative measures, such as immunization against the main carcinogenic Human Papillomavirus (HPV) subtypes and screening, particularly with methods based on HPV detection [2]. Depending upon the stage of the disease, the treatment regimen is selected based on systemic chemotherapy, radiation therapy, surgery, immunotherapy or targeted therapy [3]. In total, 5% to 26% of early-stage patients who receive combination chemotherapy, radiation therapy, or surgery for CC recur after the initial course of treatment [4] and have a very variable 5-year survival rate, ranging from 15% to 50% [5]. Recently, efforts have been focused upon treatment of advanced and recurrent disease using combination strategy [3].

ONC201 (also known as TIC10 or dordaviprone) was initially identified as a first-in-class small molecule that prompts cancer cell death via induction of TNF-Related Apoptosis-Inducing Ligand (TRAIL) [6]. It was shown to inactivate Akt and Erk thereby retaining the activity of Foxo3a, which then transcriptionally activates TRAIL expression [7]. Later, it was shown that ONC201 induces endoplasmic reticulum (ER) stress response or integrated stress response (ISR) in various cancer types, which culminate through ATF4 activation. ATF4 induces expression of death receptor DR5 and eventually cell death [8,9]. ONC201 also functions as an allosteric agonist of caseinolytic protease (clpP), a mitochondrial serine protease found in the matrix of the mitochondria [10,11]. Hyperactivation of cIpP leads to impaired oxidative phosphorylation and death of tumor cells [12]. Thus, ONC201 treatment ultimately leads to TRAIL-dependent and TRAIL-independent apoptosis in various types of cancer cells. It has shown wide-range efficacy in preclinical models of solid and haematological cancers alone and in combination with chemotherapy, targeted therapy as well as radiotherapy [13,14,15,16,17,18]. ONC201 has been extraordinarily safe as the pharmacokinetic (PK), efficacy and pharmacodynamic (PD) outlines in Phase I and II clinical trials are very promising [19,20]. Yet, there is no report on the potency of this versatile drug against cervical cancer. In the present study, we sought to test the in vitro efficacy and mechanism of action of ONC201 in HPV-positive cervical carcinoma cells.

## 2. Materials and Methods

### 2.1. Cell Culture and Reagents

Human cervical cancer cell lines HeLa (HPV 18+) and SiHa (HPV 16+) were obtained from ATCC (Rockville, MD, USA). Both cell lines were cultured in HyClone Dulbecco’s Modified Eagle Medium (DMEM) (high glucose with L-glutamine and sodium pyruvate) (Cytiva, Wilmington, DE, USA) containing 10% fetal bovine serum (FBS) (Gibco, Thermo Fisher Scientific, Waltham, MA, USA) and 1X antibacterial-antimycotic solution (Gibco). Cells were maintained at 37 °C, 5% CO_2_ in a humidified incubator. ONC201 (TIC10), doxorubicin and gemcitabine were purchased from Sigma (St. Louis, MO, USA). To prepare stock solutions, ONC201 was dissolved in dimethyl sulfoxide (DMSO) (for 100 mM stock) and doxorubicin and gemcitabine were dissolved in distilled water (for 10 mM stocks). Aliquots of stock solutions were stored at 4 °C for ONC201 and at −20 °C for doxorubicin and gemcitabine until use.

### 2.2. Cytotoxicity Assay

The cytotoxic potential of ONC201 was evaluated on cervical cancer cell lines using MTT assay [21]. Cells were seeded at 3000 cells/well in a 96-well plate and treated with different concentrations of ONC201 for 72 h or 96 h. At the end of treatment, MTT reagent (Sigma) was added at a final concentration of 0.5 mg/mL to each well. After incubation for 4 h, 100 µL of DMSO was added to each well to dissolve formazan crystals and absorbance was measured at 570 nm using Synergy H1 BioTek plate reader (Agilent Technologies, Santa Clara, CA, USA). Data were analyzed to determine the IC50 (concentration of drug that inhibited cell growth by 50%) using GraphPad Prism software (version 8).

### 2.3. Clonogenicity Assay

HeLa and SiHa cells were plated in 6-well plates at densities of 300 and 500 cells/well, respectively. After overnight incubation, cells were treated with ONC201 for 48 h. Medium with ONC201 was then replaced with culture medium and plates were further incubated for 10–14 days. Culture media in the wells were changed every two to three days depending on the metabolic rate of the cell line. Once visible colonies appeared, cells were fixed using methanol:glacial acetic acid (1:2), and 0.5% crystal violet was used to stain the colonies. Plates were rinsed with distilled water and dried, and then images were captured and the no. of colonies were counted manually.

### 2.4. Cell Cycle Analysis

Cell cycle analysis of CC cells was performed using flow cytometry as previously described [22,23]. Briefly, cells were seeded at a density of 0.6 × 10^6^ in 6 cm^2^ plates. The next day, cells were treated for 48 h and 72 h with different doses of ONC201. At the end of the treatment period, cells were washed with PBS once and fixed in ice-cold 70% ethanol overnight at −20 °C. Cells were then washed with PBS twice and resuspended in 500 µL PBS containing 10 µg/mL of RNase A (Sigma) and 20 µg/mL of propidium iodide (PI) (Sigma). After incubation at room temperature (RT) for 30 min in the dark, cells were analyzed by flow cytometry using BD FACS Aria (BD Biosciences, San Jose, CA, USA) at IIT Bombay, Mumbai, India. Data were analyzed using the free online software Floreada.io (https://floreada.io/) (accessed on 1 June 2024).

### 2.5. Annexin-V/PI Assay

Annexin-V/PI assay was performed for detecting apoptosis as previously described [24]. For this assay, HeLa and SiHa were seeded in 6 cm^2^ cell culture dishes at a density of 0.6 × 10^6^ cells per dish. The next day, cells were treated for 48 h with ONC201. Harvested cells were washed with PBS twice, resuspended in 1X Annexin Binding Buffer and stained with annexin-V-FITC and PI (Thermo Fisher Scientific). Cells were analyzed by flow cytometry using BD FACS Aria^TM^ within 15 min (BD Biosciences) at IIT Bombay, Mumbai, India.

### 2.6. Caspase-Glo^®^ 3/7 Assay

Caspase-3/7 enzyme activity was measured in ONC201-treated CC cells as previously described [25]. Briefly, HeLa (2000 cells/well) and SiHa (2500 cells/well) were seeded in 384-well white bottom plates and incubated overnight at 37 °C in a 5% CO_2_ incubator. The next day, cells were treated with ONC201 for 24 h. After the treatment, 25 µL of Caspase-Glo^®^ 3/7 reagent (Promega, Madison, WI, USA) was added to each well and incubated at RT for 1 h. Luminescence was measured on a BioTek Synergy microplate reader (Agilent Technologies).

### 2.7. Acridine Orange/Ethidium Bromide (AO/EB) Assay

HeLa and SiHa cells were seeded into 6-well plates and incubated overnight. Cells were then treated with ONC201 for 48 h. After 48 h of treatment, a dual fluorescent staining solution containing 100 µg/mL acridine orange and 100 µg/mL ethidium bromide (both fluorescent dyes purchased from SRL, Mumbai, India) was added to each well. Morphology of untreated and ONC201-treated cells was observed using Vert. A1 Axio vision inverted fluorescence microscope (Carl Zeiss AG, Jena, Germany) [26].

### 2.8. Real-Time PCR

HeLa and SiHa cells were seeded at 0.9 × 10^6^ cells per dish in 6 cm^2^ culture dishes. Post 24 h of seeding, cells were treated with ONC201 for 24 h and 48 h. Cells were harvested and total RNA was extracted using TRIzol^TM^ Reagent (Thermo Fisher Scientific). iScript cDNA Synthesis Kit (Bio-rad, Hercules, CA, USA) was used to convert mRNA into cDNA. Synthesized cDNA was used to perform quantitative real-time PCR (qRT-PCR) using PowerUp^TM^ SYBR^TM^ Green Master Mix (Thermo Fisher Scientific) on StepOne^TM^ Real-Time PCR System (Thermo Fisher Scientific) using the following conditions: initial denaturation at 95 °C for 2 min, followed by 35 continuous cycles of denaturation at 95 °C for 30 s, annealing at primer-specific temperatures for 30 s and extension at 72 °C for 30 s. The final extension step was performed at 72 °C for 7 min. The specificity of the primers was confirmed using a melt curve analysis, the 2^−ΔΔCt^ method was employed to obtain the relative quantification values, and 18s rRNA was used as a housekeeping control for normalization. The primers’ nucleotide sequences and annealing temperatures are given in Table S1.

### 2.9. Western Blot Analysis

HeLa and SiHa cells were seeded at a density of 1.2 × 10^6^ cells/dish in 6 cm^2^ culture dishes. After overnight incubation, cells were treated with ONC201 for 24 h and 48 h. Post treatment, cells were scraped in 1X PBS using a cell scraper, once again washed with 1X PBS and lysed with RIPA lysis buffer, supplemented with cOmplete^TM^ protease inhibitor (Sigma) and PhosSTOP^TM^ phosphatase inhibitor (Sigma). Post incubation on ice for 30–45 min, cell lysates were centrifuged at 13,000 rpm for 5 min at 4 °C to pellet down the debris, supernatants were collected in fresh 1.5 mL vials. Protein estimation was performed using Bradford reagent (Sigma) and BioTek Synergy microplate reader. Protein samples were prepared for loading by mixing with 4X Laemmli buffer and incubated at 95 °C for 5 min. An equal amount of total protein (30 µg) was loaded onto 10–15% SDS-polyacrylamide gel (SDS-PAGE) and electrophoresis was carried out. After SDS-PAGE, Western blotting was carried out using Trans-Blot^®^ SD semi-dry transfer apparatus (Bio-rad) wherein proteins were transferred onto a PVDF membrane. Blocking of the blot was performed with 5% non-fat dried milk (NFDM) made in 0.1% Tween-20 in 1X PBS (PBST) for 1 h at RT followed by incubation with primary antibody overnight at 4 °C, then washed with PBST thrice (3 min each) and then incubated with HRP-conjugated secondary antibody for 1.5 h at RT. The blots were imaged using Bio-Rad Molecular Imager^®^ ChemiDoc XRS+ System with Image Lab™ Software version 5.2.1 and Enhanced Chemiluminescence (ECL) substrate (Thermo Fisher Scientific). Densitometric analysis was performed using ImageJ software.

### 2.10. In Vitro Combination Assay

Cells were seeded in 96-well plates and treated with standard drug and ONC201 using two strategies: sequential and simultaneous combination. In sequential combination, a range of concentrations of a standard drug (viz. doxorubicin or gemcitabine) was added for 24 h and then replaced with ONC201 for the next 72 h. For simultaneous combination, both the drugs (i.e., the standard drug and ONC201) were added together for a period of 96 h. In both strategies, each concentration of the standard drug and ONC201 were combined in a fixed ratio depending upon their IC50 values. The combination index (CI) was calculated using CompuSyn software wherein CI < 1, =1, and >1 indicate synergism, additive effect and antagonism, respectively [27].

### 2.11. Statistical Analysis

Graphs were plotted as mean ± standard deviation (SD) from three biological replicates using GraphPad Prism software (version 8). Statistical significance was calculated between two groups (control and treated) using Student’s unpaired *t*-test. Bonferroni multiple comparison test was applied for correction of *p* values. (* *p* < 0.05, ** *p* < 0.01, *** *p* < 0.001).

## 3. Results

### 3.1. ONC201 Exerts Cytotoxicity in Cervical Cancer Cell Lines

**Figure 1:**
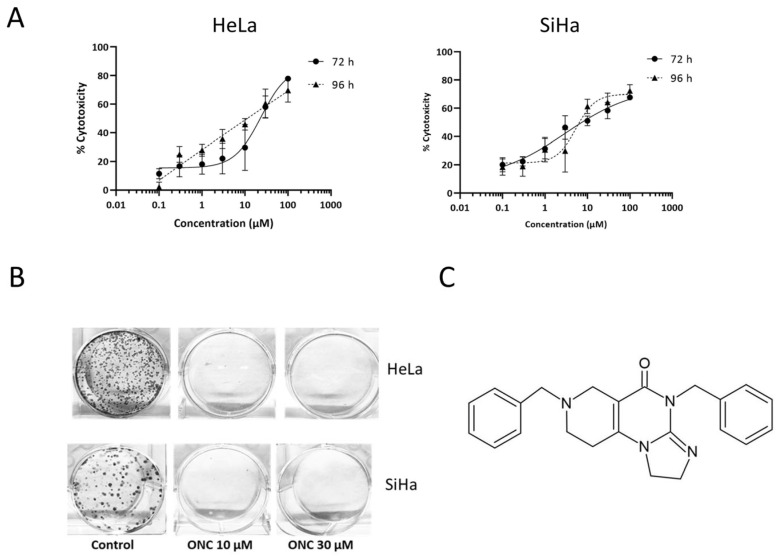
ONC201 exerts cytotoxicity in cervical cancer cell lines. (**A**) Cytotoxic potential of ONC201 against HeLa and SiHa cell lines after 72 h and 96 h of treatment. (**B**) ONC201 significantly inhibits clonogenic potential of CC cell lines (ONC: ONC201). (**C**) Chemical structure of ONC201.

ONC201 exhibited dose- and time-dependent cytotoxicity in both cell lines (Figure 1A) with IC50 values of 23.6 ± 2 µM for HeLa and 16.3 ± 0.3 µM for SiHa post 72 h of treatment (Table 1).

A clonogenicity assay was performed to assess the capability of treated cells to multiply in vitro over the long term post ONC201 treatment [28]. The colony-forming ability of CC cells was completely abrogated for the cells treated with the optimal and suboptimal doses of ONC201 as compared to the untreated control (Figure 1B). ONC201 at 3 µM concentration did not affect the colony-forming potential in both cell lines. The chemical structure of ONC201 is shown in Figure 1C.

### 3.2. ONC201 Induces S/G2-M Arrest in CC Cells

**Figure 2:**
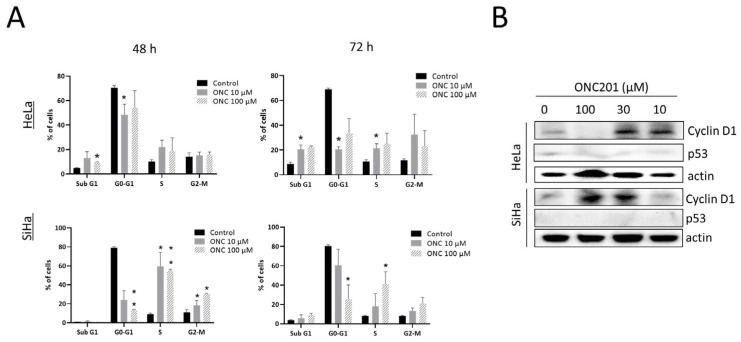
ONC201 induces S/G2-M phase arrest and increases expression of cyclin D1 in CC cells. (**A**) Cell cycle analysis of CC cells after 48 h and 72 h of treatment with ONC201 (percent population of cells in each phase). (**B**) ONC201 increases expression of cyclin D1 in both CC cell lines while p53 expression remains unchanged. (ONC: ONC201), * *p* < 0.05, ** *p* < 0.01.

As demonstrated by cell cycle analysis, treatment with ONC201 for 48 h and 72 h significantly increased S phase fraction in HeLa (at 72 h) and SiHa (at 48 h and 72 h) as well as G2-M phase fraction in SiHa (at 48 h) whilst reducing G0-G1 fraction in both the CC cell lines (Figure 2A). A significant sub G1 fraction indicating cell death was observed only in HeLa cells at both timepoints. Representative cell cycle histrograms are shown in Figure S1.

In view of the observed cell cycle arrest, cyclin D1 and p53 protein levels were examined in ONC201-treated cells by protein expression analysis. To our surprise, over a 48 h treatment period, ONC201 enhanced the expression of cyclin D1 in HeLa (at 10 µM and 30 µM, *p* < 0.05) and SiHa (at 30 µM and 100 µM, *p* < 0.01) in spite of the observed inhibition of proliferation in cell cycle studies. Whilst p53 levels remained unchanged in response to ONC201 treatment (Figure 2B), our findings indicate that ONC201 induces S/G2-M arrest accompanied by upregulated cyclin D1 expression particularly in CC cells. Densitometric analysis of all Western blots is given in Figure S2.

### 3.3. ONC201 Induces Integrated Stress Response While Downregulating Akt and Erk Phosphorylation

**Figure 3:**
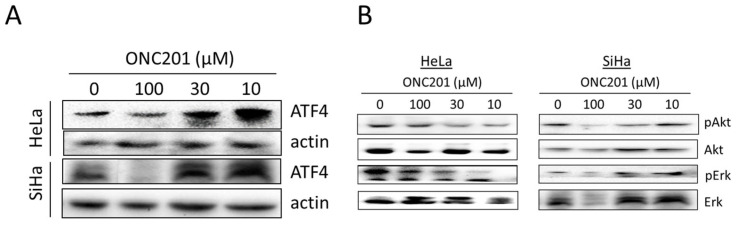
(**A**) ONC201 upregulates expression of ATF4–a marker for integrated stress response and (**B**) changes phosphorylation levels of Akt (pAkt) and Erk (pErk) in CC cell lines with varied potency (24 h treatment).

In the present study, we observed that ONC201 upregulates ATF4 protein levels at 10 µM and 30 µM in both CC cell lines (*p* < 0.01) (Figure 3A). In addition, the drug was found to downregulate phosphorylation of its well-known targets viz. Akt and Erk significantly in HeLa cells only. Unexpectedly, it upregulated phospho-Erk in SiHa cells at 10 µM and 30 µM (Figure 3B).

### 3.4. Apoptosis Is Induced by ONC201 in CC Cells

**Figure 4:**
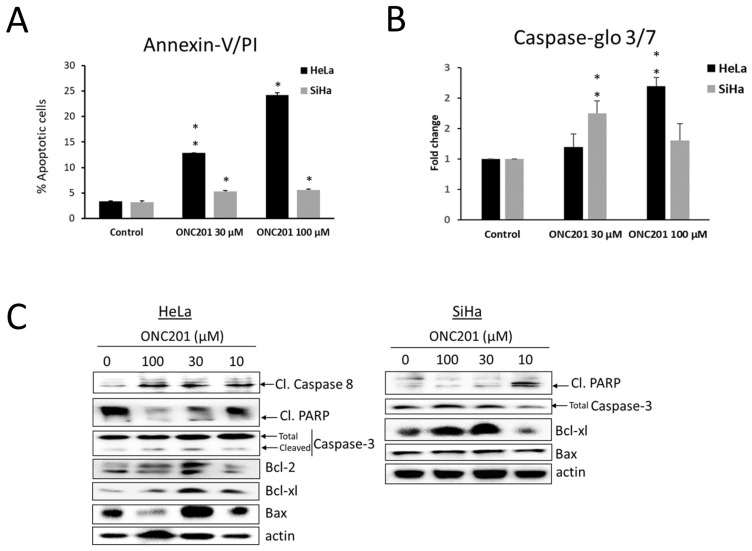
(**A**) ONC201 induces significant apoptosis in HeLa and SiHa cells after 48 h treatment. (**B**) An increase in caspase-3/7 enzymatic activity is seen upon ONC201 treatment in both cell lines. (**C**) ONC201 induces PARP cleavage and Bax upregulation in both cell lines while cleaved caspase-3, 8 are observed only in HeLa upon ONC201 treatment. Basal levels of Bcl-2 and caspase-8 are undetectable in SiHa cells. Bcl-xl is significantly upregulated by ONC201 in both cell lines. * *p* < 0.05, ** *p* < 0.01.

After observing cell cycle abrogation and increased sub G1 fraction in ONC201-treated CC cells, apoptosis-inducing potential of ONC201 was confirmed by Annexin-V/PI assay [29]. After 48 h of ONC201 treatment, this assay demonstrated a significant increase in no. of apoptotic cells in both cell lines (*p* < 0.05) (Figure 4A, dot plots are shown in Figure S1).

Further, Caspase-Glo^®^ 3/7 assay was used to confirm the increase in caspase-3/7 activity (a hallmark of apoptosis) upon drug treatment. ONC201 significantly increased caspase-3/7 activity in both cell lines (in HeLa - 2.2 fold at 100 µM and in SiHa - 1.8 fold at 30 µM) (Figure 4B). In both of the above-mentioned assays, it was observed that HeLa was more sensitive to ONC201 as compared to SiHa. We also confirmed ONC201-induced apoptosis in both cell lines by dual staining with acridine orange and ethidium bromide (AO/EB) and fluorescence microscopy (Figure S3).

After confirming apoptosis by the above-mentioned cell-based assays, the effect of ONC201 was tested on the expression of apoptotic markers, i.e., cleaved PARP, cleaved caspase-3 and Bcl-2 family proteins (viz. Bcl-2, Bcl-xl and Bax) (Figure 4C). The results of immunoblotting showed that ONC201 increased the expression of cleaved PARP in both cell lines when compared to untreated control, confirming that ONC201 induces apoptotic pathway in CC cells. Additionally, HeLa cells treated with ONC201 showed an increase in cleaved caspase-3 and cleaved caspase-8 levels.

Bcl-2 family proteins such as Bcl-2, Bcl-xl (anti-apoptotic) and Bax (pro-apoptotic) play key roles in regulating mitochondrial membrane permeability, mitochondrial function and cytochrome c release. After 48 h of treatment with ONC201, Bcl-2 levels showed no significant change but Bax was increased significantly by 30 µM ONC201 in both the CC cell lines. Unexpectedly, Bcl-xl was observed to be upregulated by ONC201 in both cell lines significantly at 30 µM and 100 µM.

### 3.5. ONC201 Upregulates Pro-Apoptotic Gene Expression

**Figure 5:**
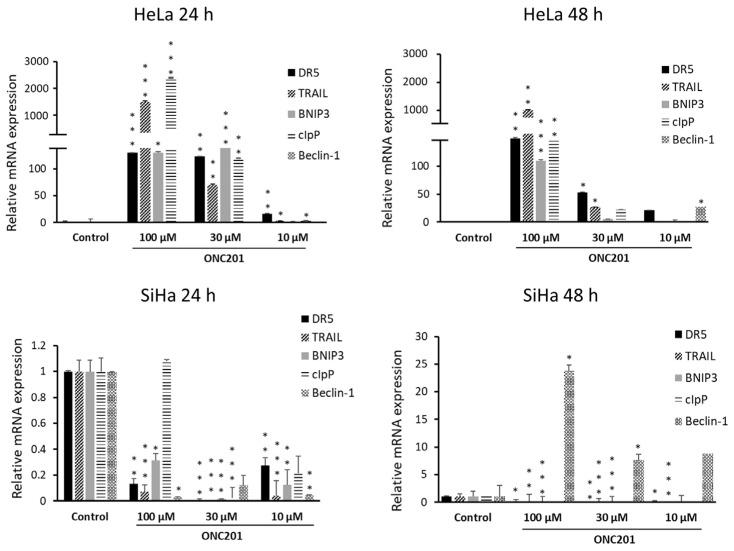
ONC201 induces expression of its well-known targets viz. DR5, TRAIL and cIpP in HeLa (**top panel**) while it downregulates these genes in SiHa (**bottom panel**). Autophagy-related genes viz. BNIP3 and Beclin-1 are also significantly upregulated by ONC201. * *p* < 0.05, ** *p* < 0.01, *** *p* < 0.001.

In the present study, we observed significant upregulation of TRAIL, DR5 and cIpP mRNA expression in HeLa but not in SiHa cells in spite of ONC201-induced apoptosis observed in both of these cell lines (Figure 5). In addition, autophagy-related proteins viz. BNIP3 and Beclin-1 were upregulated in response to ONC201-BNIP3 in HeLa (at 24 h with 30 µM and 100 µM, at 48 h with 100 µM) and Beclin-1 in SiHa (at 48 h with 30 µM and 100 µM). A significant increase in Beclin-1 levels was also evident in HeLa after 48 h of treatment with ONC201 (at 10 µM and 100 µM). Overall, these results suggest that these cells possibly witness upregulation of autophagy in response to all or at least specific dose/s of ONC201. At the same time, clearly, ONC201 induces both extrinsic (TRAIL-dependent) and intrinsic (TRAIL-independent) apoptosis in HeLa and only intrinsic apoptosis in SiHa.

### 3.6. ONC201 Exhibits Synergism in Combination with Standard Chemotherapeutic Agents Against CC Cells

**Figure 6:**
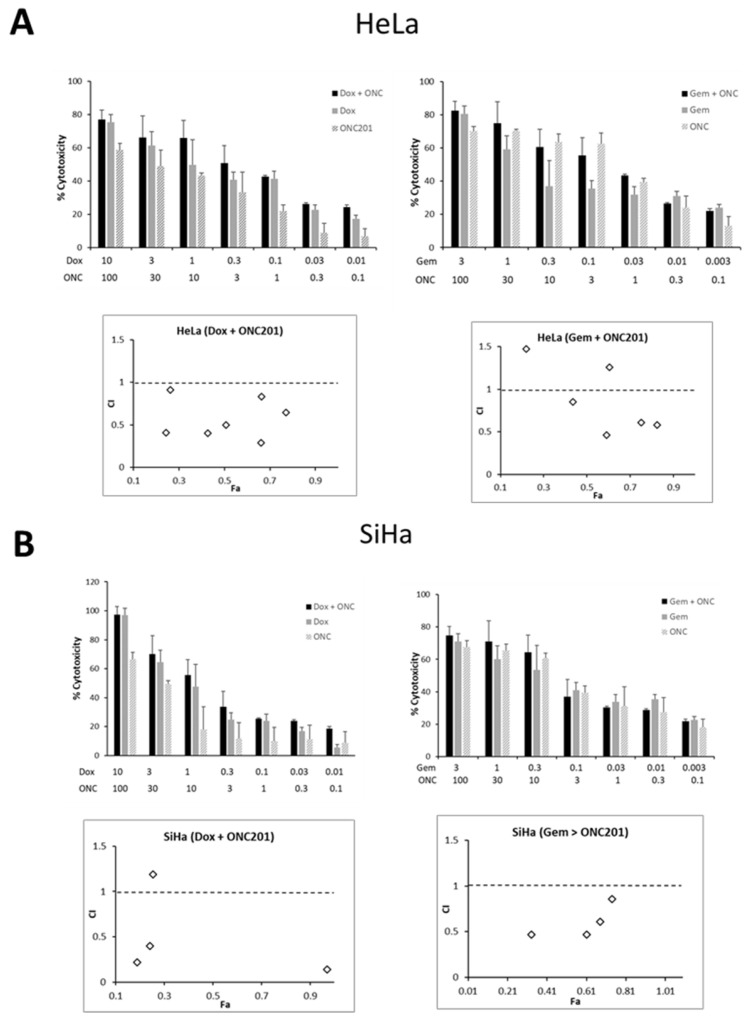
Sequential combination of ONC201 with doxorubicin or gemcitabine exhibits synergism in (**A**) HeLa and (**B**) SiHa. CI: Combination indices, Fa: Fraction affected, Dox: doxorubicin, Gem: gemcitabine, ONC: ONC201.

The efficacy of ONC201 in combination with currently used standard chemotherapeutic drugs against CC cells was tested using two approaches viz. sequential and simultaneous drug treatment [30]. It was observed that ONC201 was synergistic when applied sequentially in dual combination with doxorubicin in both CC cell lines as indicated by combination index (CI) values of <1 (Figure 6). In particular, more synergism was observed in HeLa (CI values in the range of 0.29–0.91) and moderate synergism to antagonistic effect in SiHa (CI = 0.3–2.57). Moderate synergism to antagonism was observed with a sequential combination of ONC201 and gemcitabine in HeLa (CI = 0.46–2.29) and SiHa (CI = 0.47–4.75). Among all combinations, 0.1 µM doxorubicin and 10 µM ONC201 was highly synergistic with CI = 0.29 and Fraction affected (Fa) = 0.66 in HeLa. In SiHa, 0.3 µM gemcitabine and 10 µM ONC201 was moderately synergistic (CI = 0.47, Fa = 0.61). The range of combined doses of each drug along with corresponding fraction affected and combination index values are given in Table S3. Bar graphs for simultaneous combinations in HeLa and SiHa are shown in Figure S4.

## 4. Discussion

Cervical cancer is a prevailing and lethal cancer among women in the developing world. Although ONC201 was discovered as a TRAIL-inducing drug, it is now well known that it induces integrated stress response (ISR) as an early event [8]. This response has the potential to induce cell death and is primarily coordinated by activating transcription factor-4 (ATF4) [31]. As per previous reports, TRAIL and DR5 levels increase in response to ONC201-induced ISR in addition to abrogation of Akt/Erk signaling by this versatile drug [8,11]. Previous studies have reported that CC cells are sensitive to TRAIL-induced apoptosis [32]. In the present study, ONC201 induced ATF4 expression and apoptosis in both cell lines, though it increased expression of key target genes such as TRAIL, DR5 and cIpP only in HeLa but not in SiHa cells. Also, it inhibited Akt and Erk phosphorylation effectively only in HeLa cells. In line with previous reports, TRAIL and DR5 expression could be induced due to inhibition of phospho-Akt and phospho-Erk activity by ONC201 in this particular CC cell line [33].

ONC201 triggered p53-independent cell cycle arrest in the S/G2-M phase in both CC cell lines. Previous studies showed that ONC201 induces DR5 and TRAIL pathways without the need for p53 in cancer cells [34]. In uterine sarcoma cells, ONC201 increased DR5 abundance, downregulated Akt and Erk phosphorylation and induced intrinsic and extrinsic apoptotic pathways without affecting the levels of Bcl-2 [35]. In desmoplastic small round cell tumors, ONC201 induced TRAIL and DR5 expression as well as cell death through the extrinsic apoptotic route [36]. However, another study reported that ONC201 causes cell death through both TRAIL-dependent and TRAIL-independent mechanisms in breast cancer [37]. In line with these findings, one more study showed that cytotoxicity of ONC201 is independent of both caspase cascades and death receptors in two gynecological cancers viz. breast and endometrial [38] as it was shown to induce death in these cancer cells by ATP depletion. Later, it was reported that ONC201 induces cell death via induction of cellular stress mechanisms viz. endoplasmic reticulum (ER) stress and atypical integrated stress response (ISR) in solid tumors as well as hematopoietic cancers [8,39,40]. Reportedly, ONC201-induced apoptosis via TRAIL/DR5 can be at least partially attributed to ATF4 [39]. Moreover, ATF4 activation may also lead to other phenotypic responses such as TRAIL/DR5-indepdendent apoptosis or cell cycle arrest [11].

We observed significant upregulation of Bax in response to ONC201 in both cell lines which has been proposed to be mediated by ATF4/CHOP pathway which eventually leads to intrinsic apoptosis [41]. Yet, we observed TRAIL and DR5 induction along with cleaved caspase-8, which are markers of extrinsic apoptotic in HeLa but not in SiHa cells. Overall, these results indicate that ONC201 induces both extrinsic and intrinsic apoptotic pathways in HeLa and only intrinsic apoptosis in SiHa cells. Earlier, ONC201 has been shown to induce only intrinsic apoptosis via ATF4 without caspsase-8 activation in haematological cancers [39]. In line with previous reports by others, basal levels of caspase-8 and Bcl-2 proteins were undetectable in SiHa cells [42]. We did not observe any increase in cleaved caspase-3 levels in SiHa upon ONC201 treatment in spite of the increase in cleaved PARP levels. We speculate that ONC201-induced apoptosis is mediated by caspase-7 rather than caspase-3 in this particular cell line.

In our present study, though ONC201 unexpectedly induced Bcl-xl expression in both cell lines, this could not rescue the cells from apoptosis or S/G2-M arrest. The increase in Bcl-xl could be attributed to the fact that this anti-apoptotic protein has been shown to translocate to the ER upon induction of ER stress and inhibit unfolded protein response (UPR) to promote cell survival under ER stress conditions [43]. Previous reports also suggest that Bcl-2 and Bcl-xl localize to ER and inhibit autophagy by binding to Beclin-1 [44,45]. Apart from apoptotic genes, in the present study, ONC201 was found to upregulate expression of BNIP3 and Beclin-1 in CC cell lines, which are well-known regulators of autophagy. Previously, Beclin-1 has been shown to promote radiation-induced G2-M arrest in cancer cells [46]. It is interesting to note that BNIP3 signals for apoptosis or mitophagy (i.e., removal of damaged mitochondria through autophagy) depending upon its interaction with LC3 [47] and ONC201 has been shown to kill cancer cells by targeting mitochondria [38]. Recently, ONC206–a derivative of ONC201 was shown to induce cytoprotective autophagy in hepatocellular carcinoma via cIpP [48]. It will be interesting to confirm if ONC201 induces autophagy in CC cells, and if so, to further decipher whether this autophagy pathway plays cytoprotective or cytotoxic role in future studies. A study by Wang et al. (2020) demonstrated that curcumin induces G2-M arrest in SiHa cells along with autophagy and apoptosis [49].

In addition to apoptosis, activation of ATF4 and ISR by ONC201 has been shown to induce G1 phase arrest in many cancer types viz. ovarian [50], breast [37,51], endometrial [16] and pancreatic (Panc-1 cells) [52] whilst S/G2-M arrest has been reported in prostate cancer cells [18] and several pancreatic cancer cell lines [53]. In fact, most of the human pancreatic cancer cell lines exhibit S/G2-M arrest upon ONC201 treatment and do not undergo cell death. On the contrary, in the present study, observed S/G-M arrest was accompanied by apoptosis in both CC cell lines. In line with all previous studies, ONC201-induced S/G2M arrest and apoptosis in CC cells were p53-independent [6,7,8,16,39,54,55]. There is a possibility that since CC cells commonly exhibit disrupted G1/S checkpoint, these cells are arrested in S/G2-M phase by ONC201 [56]. Reportedly, sensitivity of cervical cancer cells to standard chemotherapeutic drugs depends on their p53 status [57]. The p53-independent mode of action of ONC201 would be highly desirable as p53 function is often inhibited by viral oncoproteins in HPV-positive CC.

According to previous reports, ONC201-induced cell death or growth arrest has been typically associated with a decrease in cyclin D1 expression [8,13]. In fact, cyclin D1 expression has been proposed to be a biomarker post treatment with ONC201 for breast tumors [37]. In contrast, in the current study, for the very first time, we report a significant increase in cyclin D1 levels upon ONC201 treatment in both CC cell lines. This was certainly an unexpected finding. However, according to a previous study, cyclin D1 induces UPR and ER stress-mediated apoptosis in myeloma cells [58]. From our current findings, it is very likely that cyclin D1 is also involved in ONC201-induced ISR and ER stress-induced apoptosis in CC cells.

Combination therapy is a rational strategy to increase the response and tolerability of therapeutic agents and to decrease resistance towards them [59]. Targeted agents that inhibit oncogenic signaling pathways are used in combination therapy to increase the therapeutic index with better safety [56]. Previous studies demonstrate that ONC201 is synergistic with standard drugs in several cancers, e.g., with taxanes in breast cancer [37] and non-small cell lung cancer [7], with DNA-PKC inhibitor Nu7026 in hepatocellular carcinoma [60], gemcitabine in pancreatic cancer [52], with several drugs for haematological cancers (such as cytarabine, 5-azacytidine, bortezomib) to name a few [61]. In the present study, dual combination of ONC201 with doxorubicin showed moderate synergism in both CC cell lines.

## 5. Conclusions

The present study demonstrates the anticancer activity of the versatile drug ONC201 in cervical cancer cells. This potency of ONC201 can be attributed to its ability to induce integrated stress response, S/G2-M arrest and apoptosis in CC cell lines. Interestingly, though it induces apoptosis in both cell lines, only HeLa cells exhibit a marked increase in levels of key target genes of this drug such as TRAIL, DR5 and cIpP along with cleaved caspase-8 (a marker of TRAIL-dependent, extrinsic apoptosis). In addition, we observed increase in levels of two important mediators of autophagy viz. Beclin-1 and BNIP-3 in response to ONC201 treatment. Further, ONC201 is synergistic when used combination with standard chemotherapeutic drugs in CC cell lines. This study provides a basis for potential clinical application of this first-in-class drug as single agent or in combination with conventional therapies for cervical cancer.

## Figures and Tables

**Table 1:** IC50 values for ONC201 in CC cell lines.

Timepoint	IC50 (µM)	
	HeLa	SiHa
72 h	23.6 ± 2	16.3 ± 0.3
96 h	17.8 ± 4	12.9 ± 3

Values represented as mean ± SD from three independent experiments.

## Data Availability

The data presented in this study are included in the article and Supplementary Materials. The raw data supporting the conclusions of this article will be made available by the authors upon reasonable request.

## Supplementary Materials

The following supporting information can be downloaded at: www.mdpi.com/article/10.3390/biom15040463/s1, Table S1: List of primers used for real-time PCR; Table S2: Details of antibodies; Table S3: Combination index values; Figure S1: Cell cycle histograms and annexin-V/PI apoptosis assay dot plots; Figure S2: Densitometric analysis of Western blots; Figure S3: Acridine orange/ethidium bromide staining of ONC201-treated CC cells; Figure S4: Simultaneous combination bar graphs. File S1: Original images for western blot.
