# Supplementary material for: ONC201 (Dordaviprone) Induces Integrated Stress Response and Death in Cervical Cancer Cells
Source: Biomolecules. 2025 Mar 21;15(4):463. doi: 10.3390/biom15040463 (PMC12025107; doi:10.3390/biom15040463)

**Figure S1 – Flow cytometry histograms**

**a) Cell cycle histograms**

**HeLa 48 h**

| Control | ONC201 10 µM | ONC201 100 µM |
| --- | --- | --- |
| 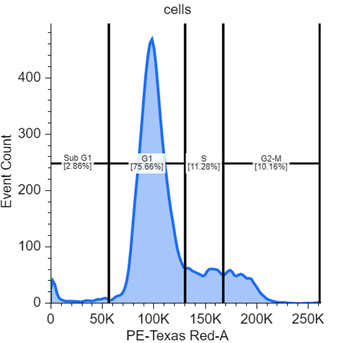 | 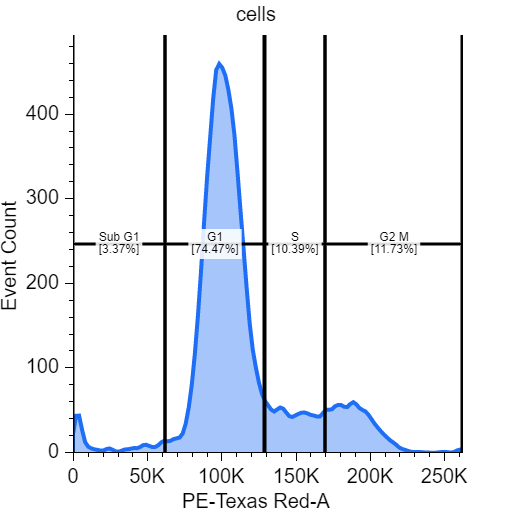 | 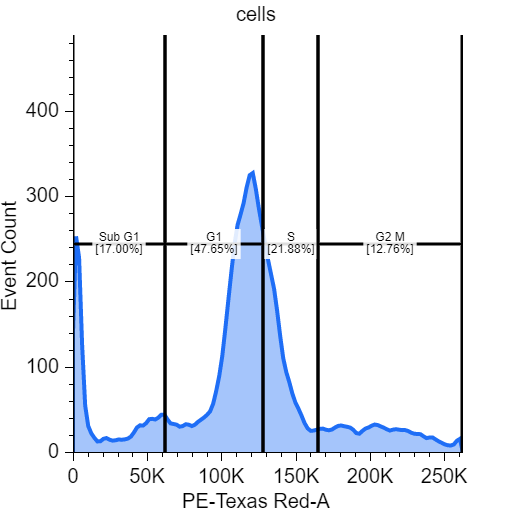 |
|  |  |  |
| **HeLa 72 h** |  |  |
| Control | ONC201 10 µM | ONC201 100 µM |
| 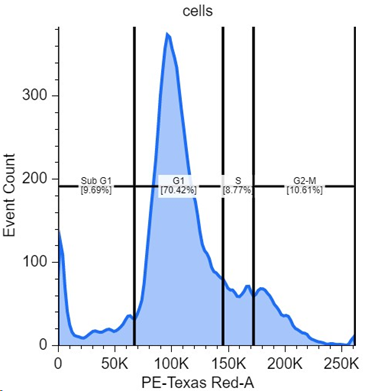 | 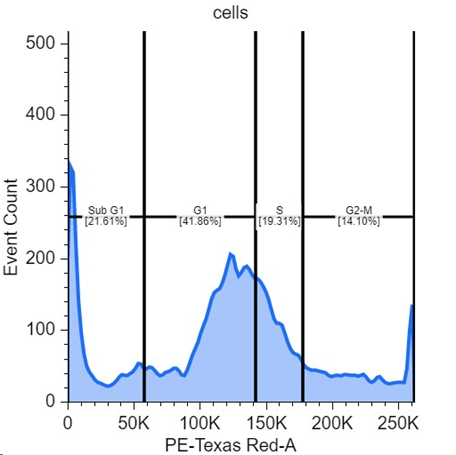 | 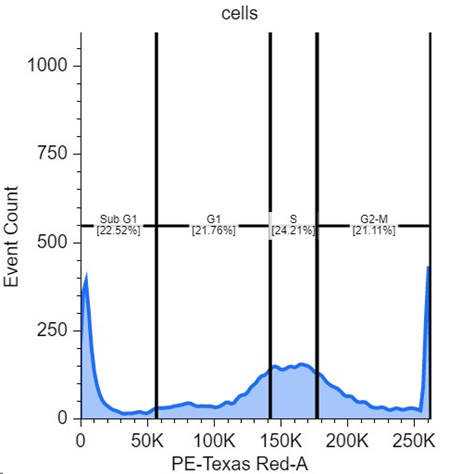 |

| **SiHa 48 h** |  |  |
| --- | --- | --- |
| Control | ONC201 10 µM | ONC201 100 µM |
| 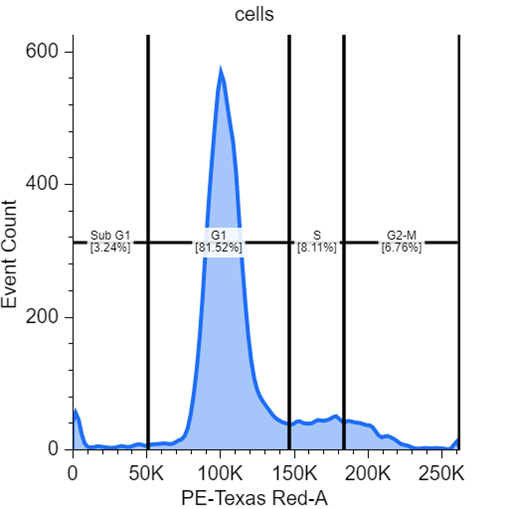 | 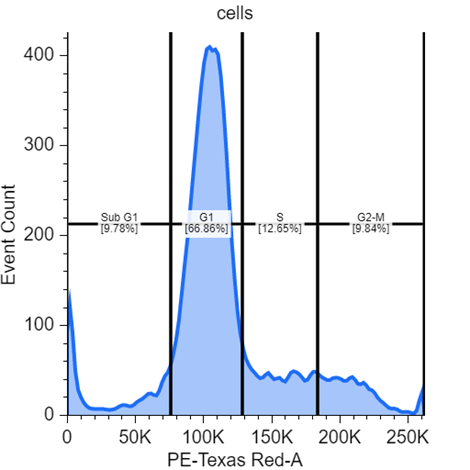 | 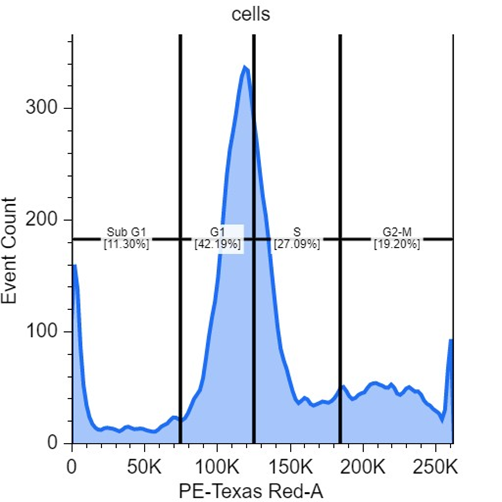 |
|  |  |  |
| **SiHa 72 h** |  |  |
| Control | ONC201 10 µM | ONC201 100 µM |
| 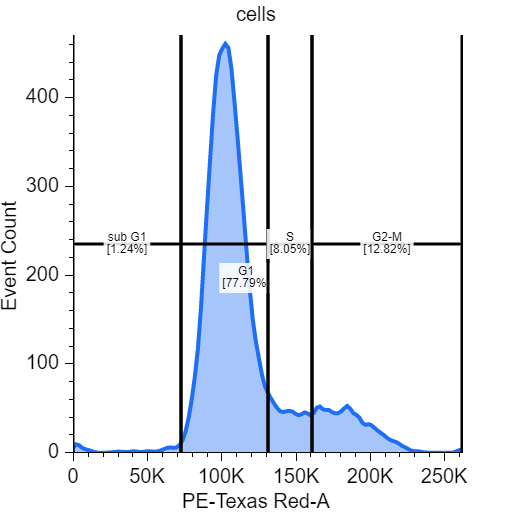 | 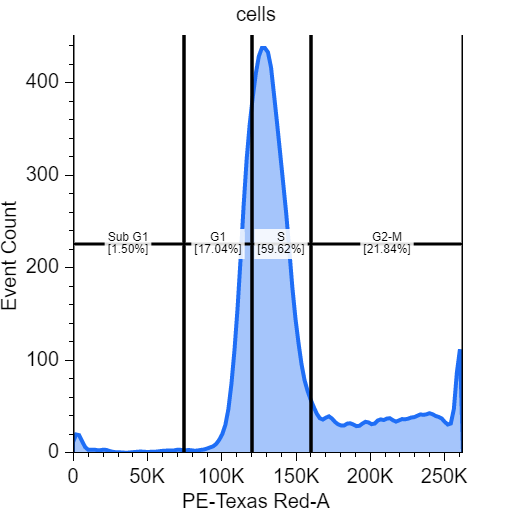 | 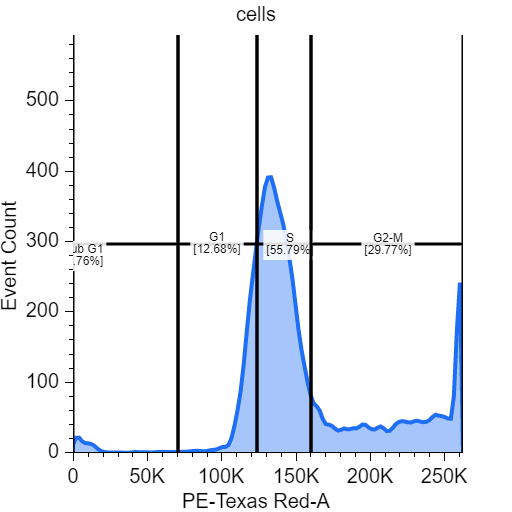 |

**b) Annexin V/PI apoptosis assay dot plots**


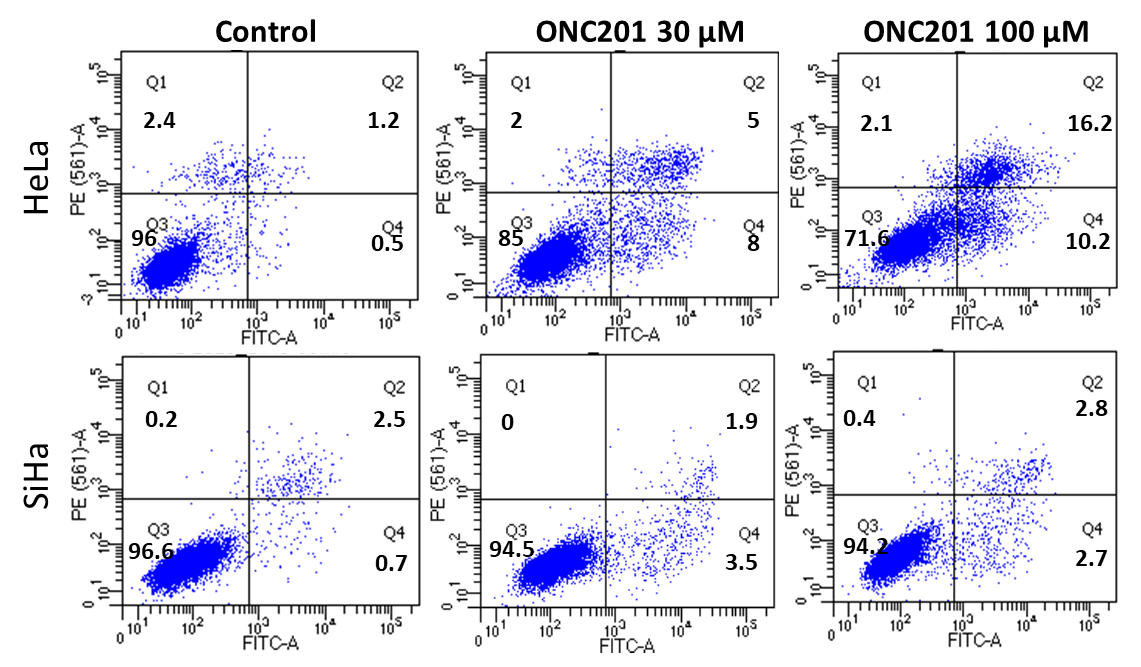

Supplement: Supplementary file 1 [file biomolecules-15-00463-s001.zip › biomolecules-3487362-supplementary new version/Figure S1 _ cell cycle histograms.docx]
