# Supplementary material for: ONC201 (Dordaviprone) Induces Integrated Stress Response and Death in Cervical Cancer Cells
Source: Biomolecules. 2025 Mar 21;15(4):463. doi: 10.3390/biom15040463 (PMC12025107; doi:10.3390/biom15040463)
Supplement: Supplementary file 1 [file biomolecules-15-00463-s001.zip › biomolecules-3487362-supplementary new version/Figure S2- Densitometric analysis for Western blots with statistics.docx]

**Figure S1 - Densitometric analysis of protein expression studies**

N=3, bars indicate mean ± SD of three independent experiments.

| **Cyclin D1, beta actin (p53 was undetectable)** |  |
| --- | --- |
| 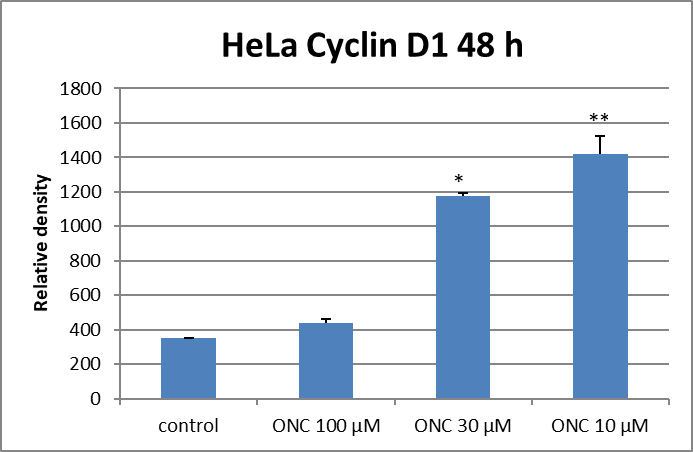 | 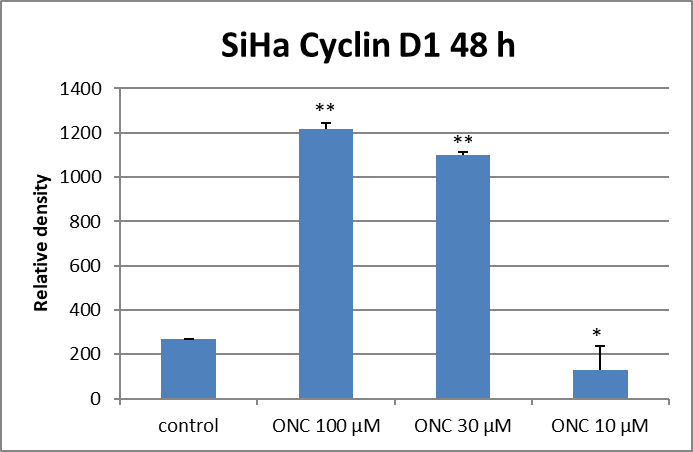 |
| 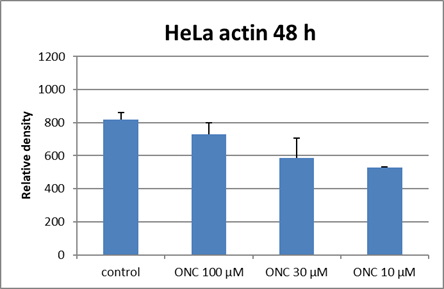 | 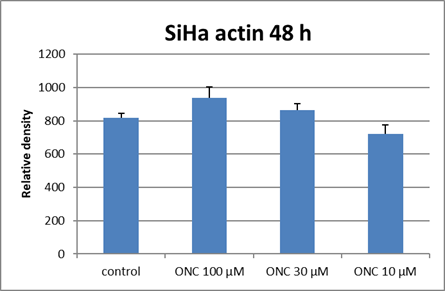 |
|  |  |
| **ATF4, pAkt, Akt, pErk, Erk**  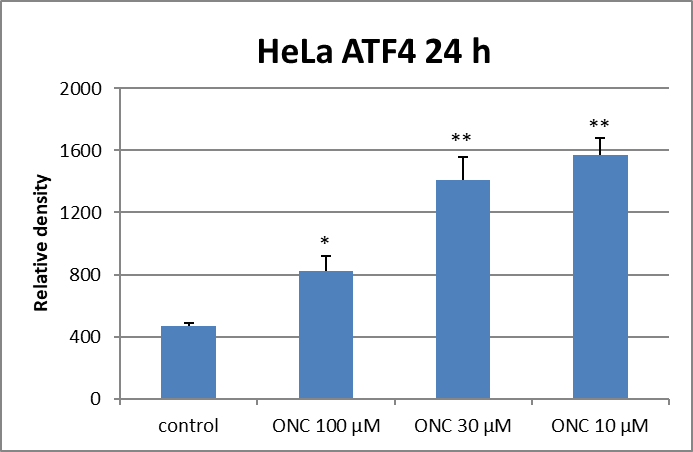 | 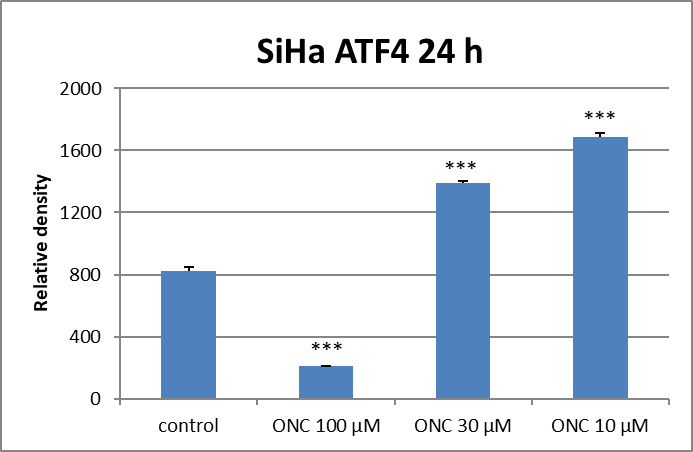 |
| 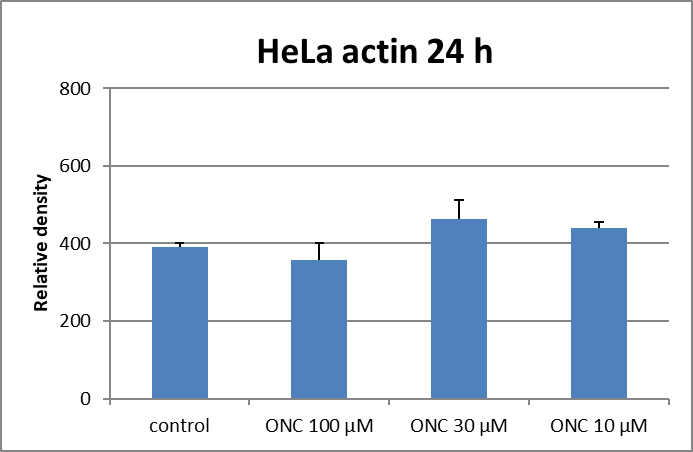 | 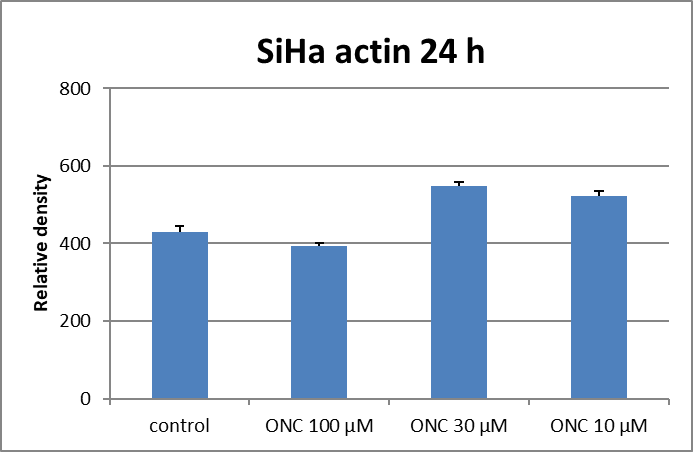 |
| 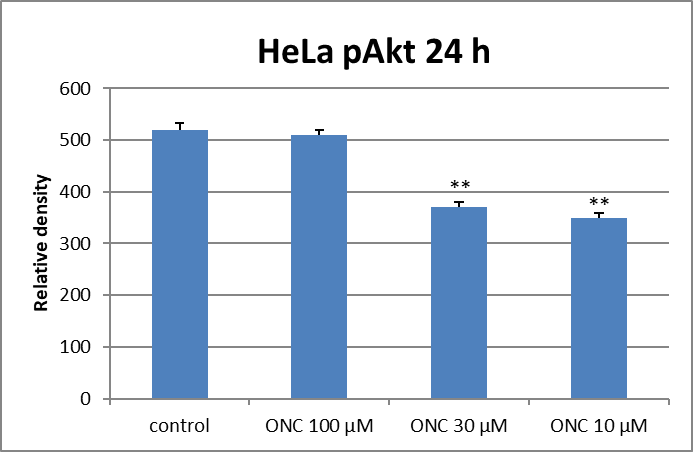 | 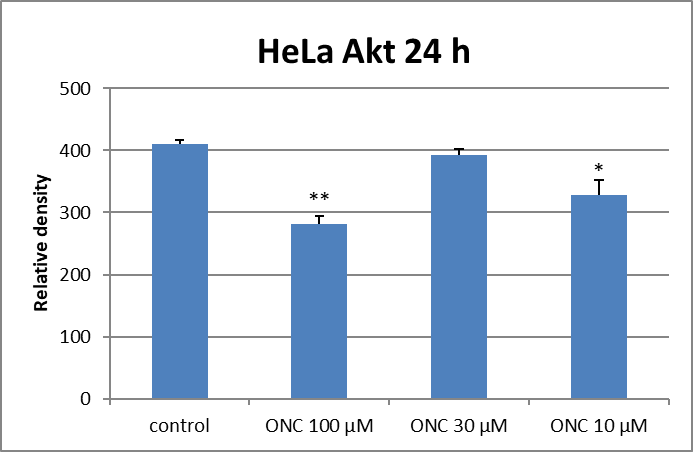 |
| 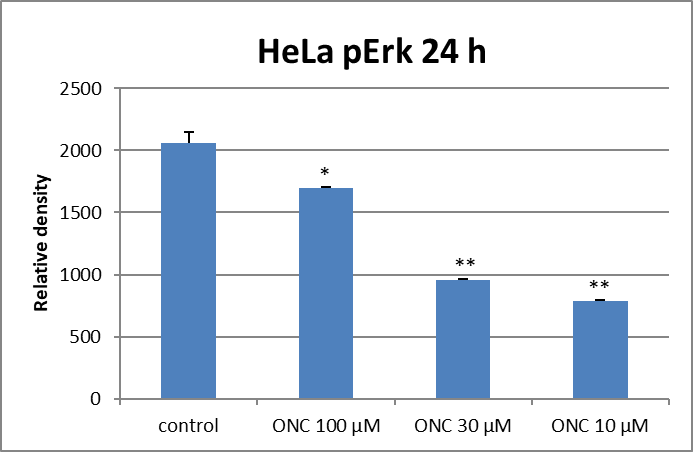 | 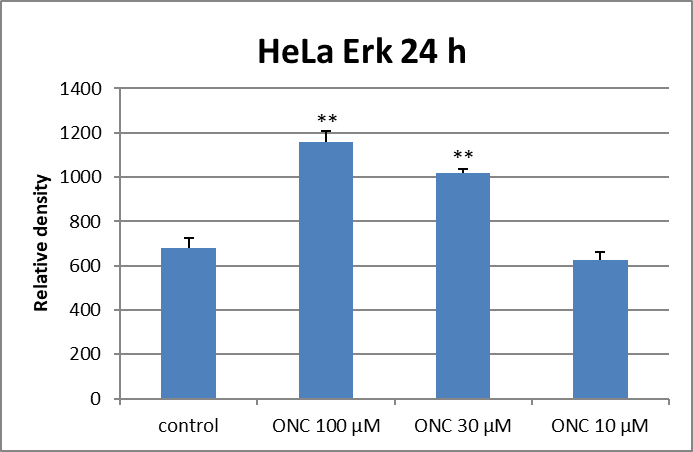 |
| 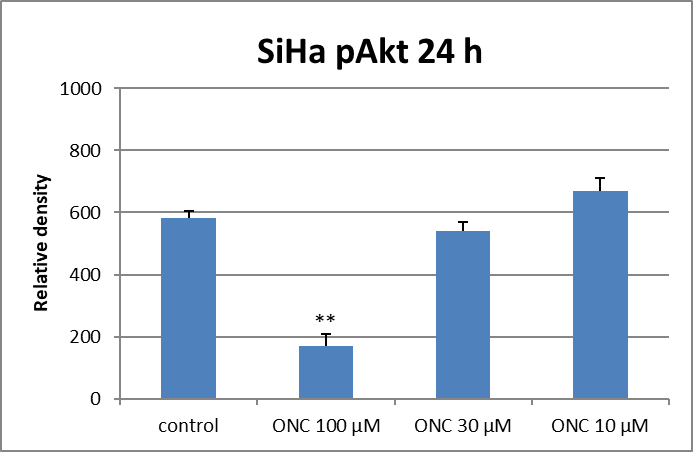 | 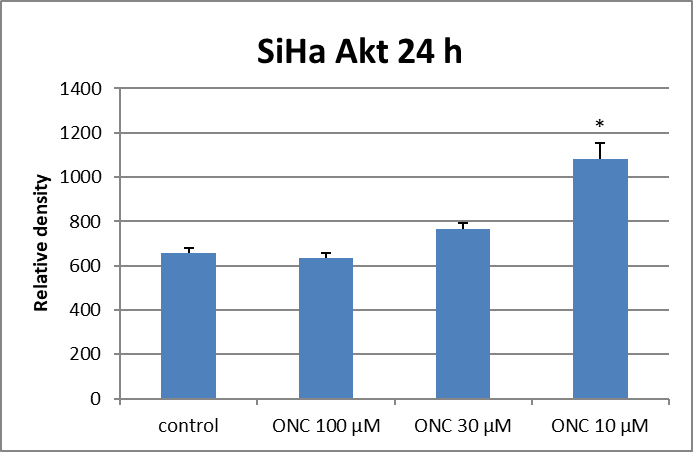 |
| 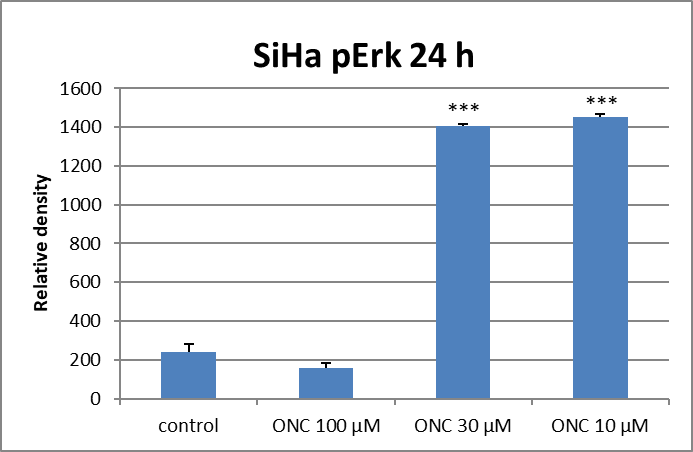 | 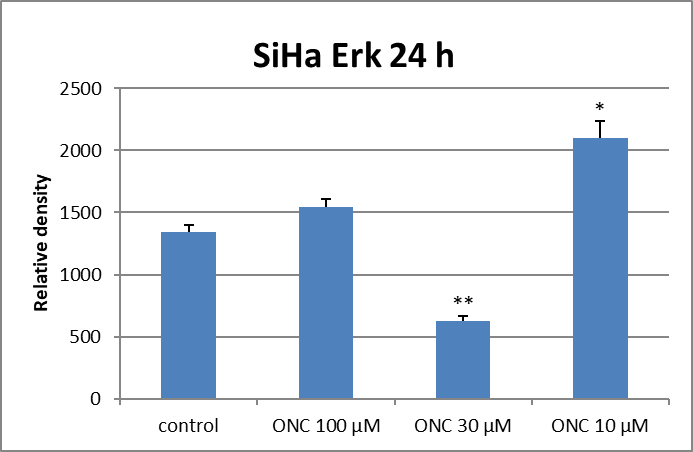 |
| **Apoptotic proteins - HeLa** |  |
| 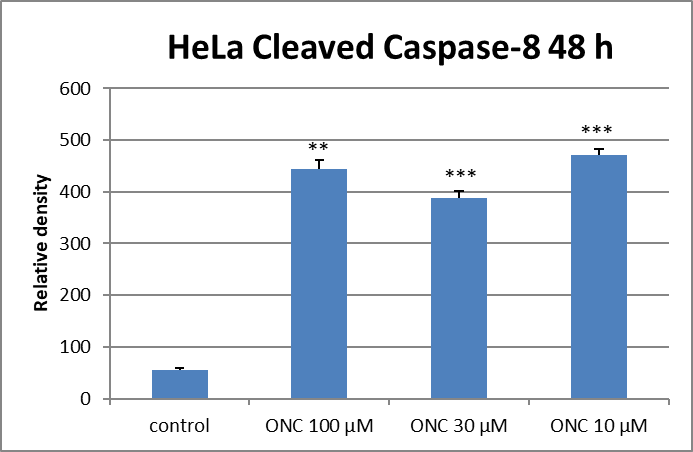 | 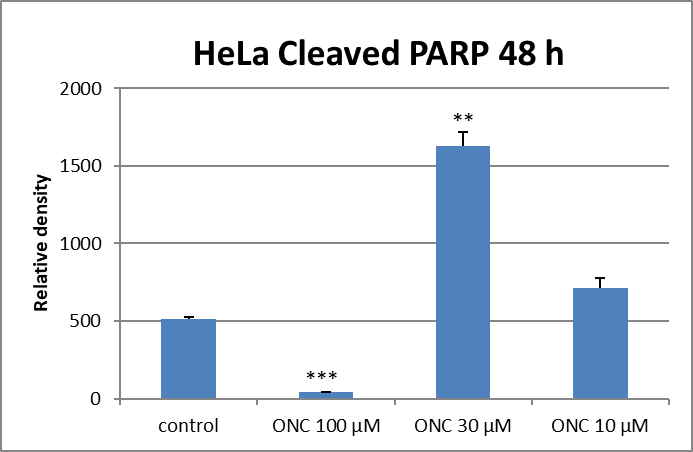 |
| 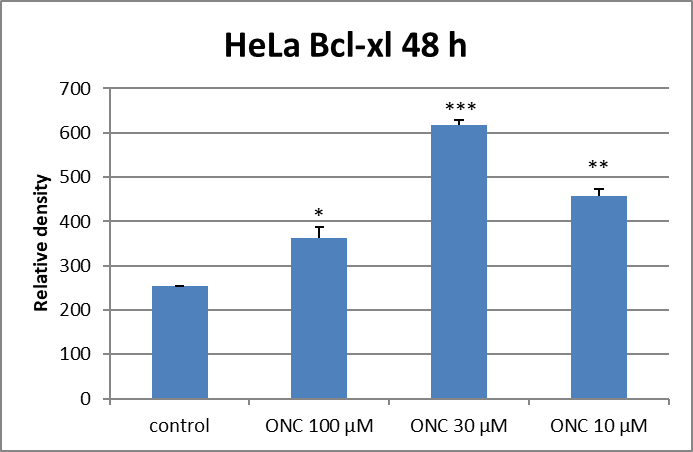 | 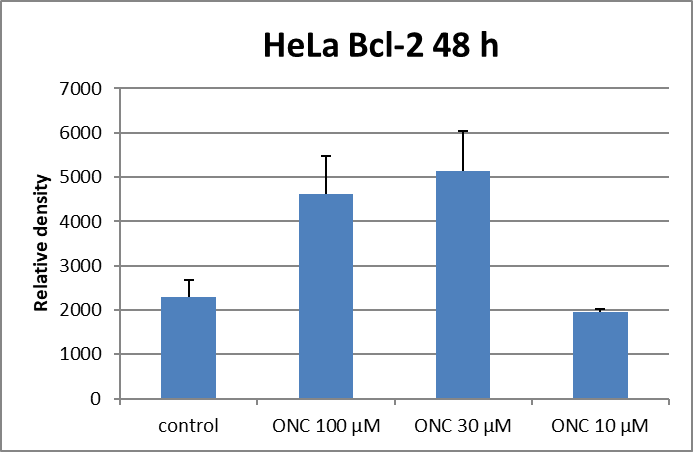 |
| 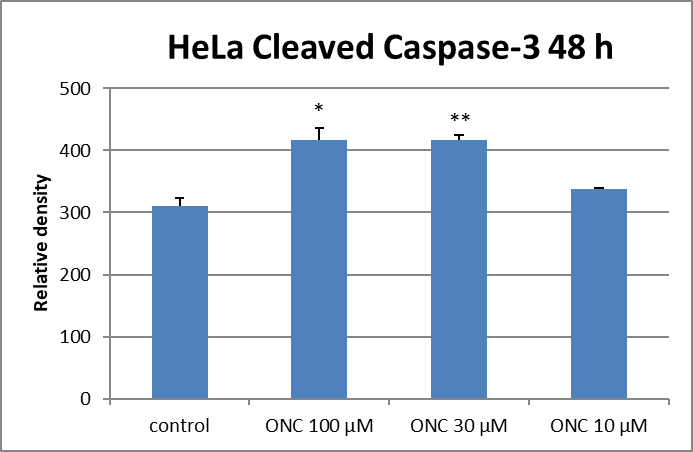 | 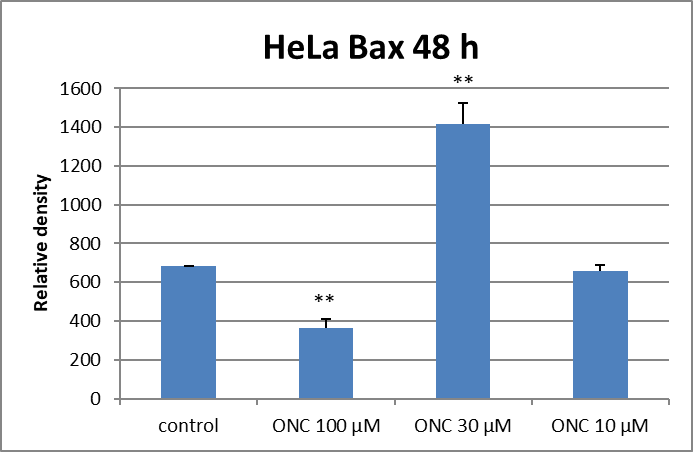 |
| 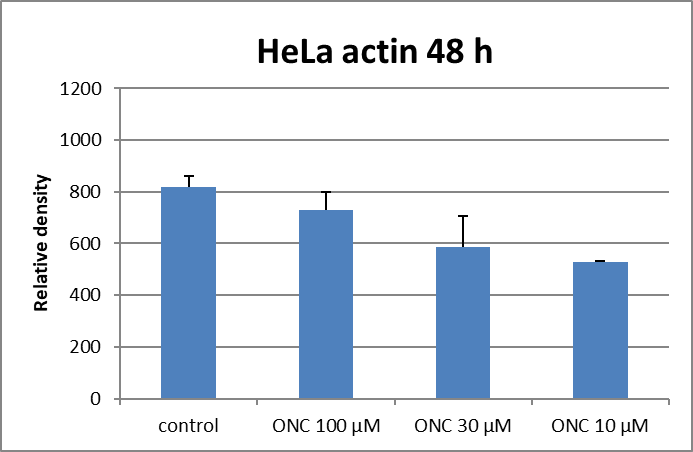 |  |
| **Apoptotic proteins - SiHa** |  |
| **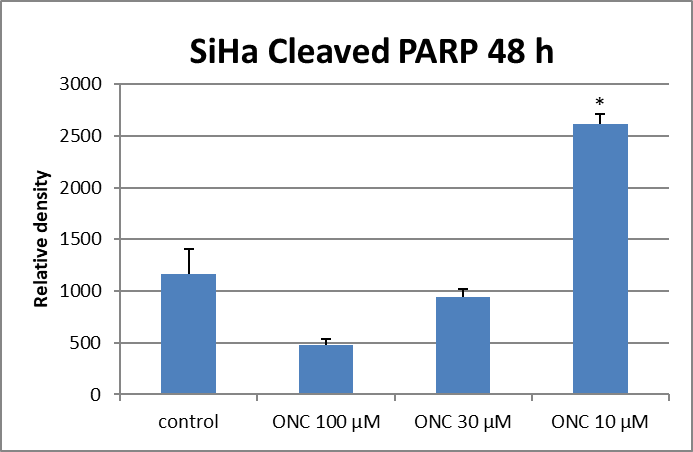** | 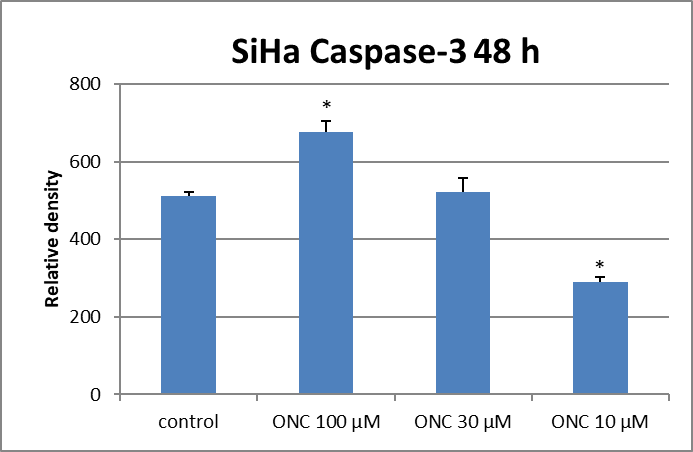 |
| **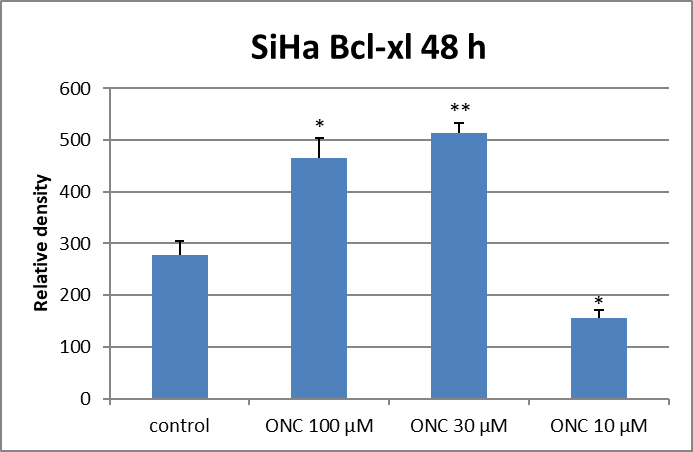** | **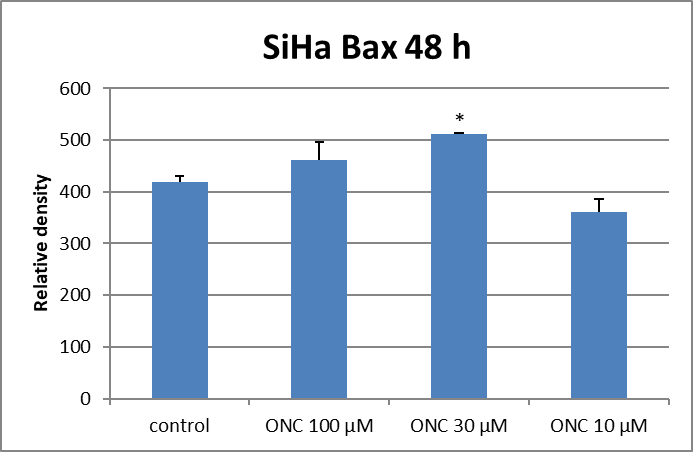** |
| **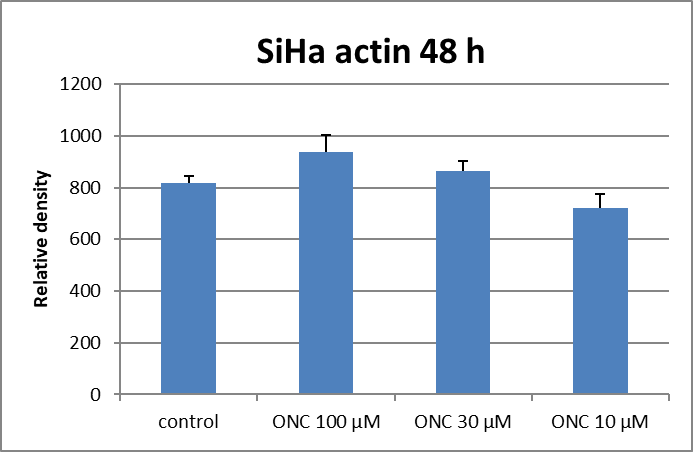** |  |
