# Supplementary material for: ONC201 (Dordaviprone) Induces Integrated Stress Response and Death in Cervical Cancer Cells
Source: Biomolecules. 2025 Mar 21;15(4):463. doi: 10.3390/biom15040463 (PMC12025107; doi:10.3390/biom15040463)
Supplement: Supplementary file 1 [file biomolecules-15-00463-s001.zip › biomolecules-3487362-supplementary new version/Figure S3- AOEB staining.docx]

**Figure S2 –**

**Acridine orange/ethidium bromide staining of ONC201-treated CC cells**


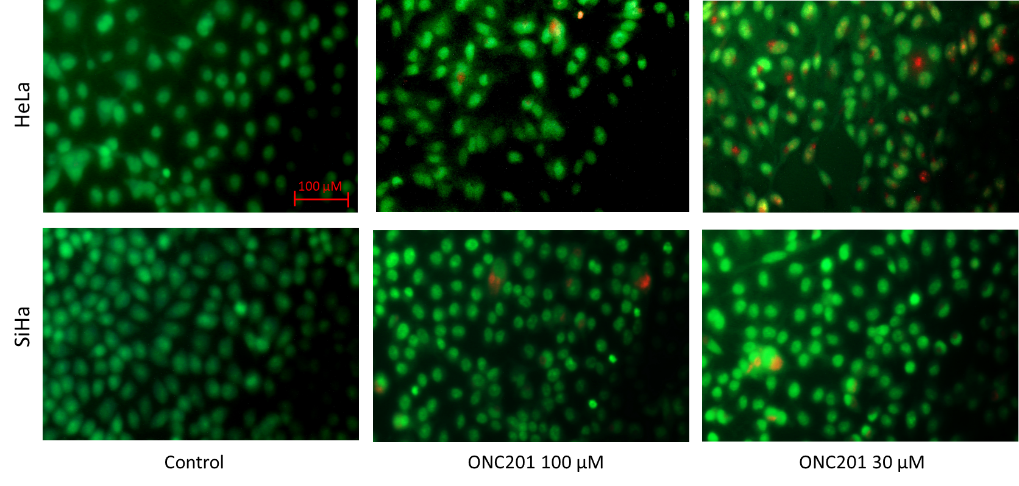


Control shows centrally located circular faint green nuclei within live cells. Early apoptotic cells (showing crescent shaped green nuclei), late apoptotic cells (with orange fluorescence) can be observed after ONC201 treatment. Some necrotic cells (with uneven red fluorescence) are observed in HeLa treated with 30 µM and in SiHa treated with 100 µM of ONC201.
