# Supplementary material for: ONC201 (Dordaviprone) Induces Integrated Stress Response and Death in Cervical Cancer Cells
Source: Biomolecules. 2025 Mar 21;15(4):463. doi: 10.3390/biom15040463 (PMC12025107; doi:10.3390/biom15040463)

**Figure S4 - Simultaneous combination bar graphs**

**a) HeLa - Dox + ONC201**

**
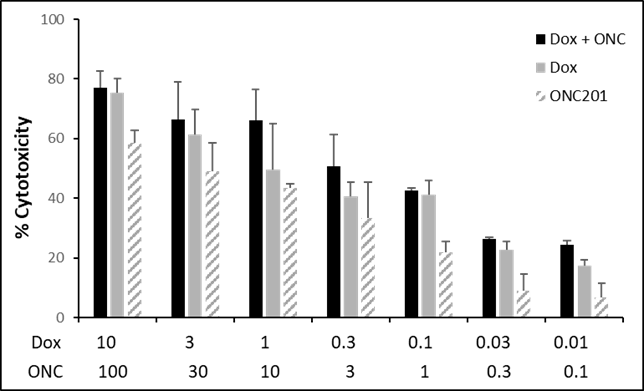
**

**b) HeLa - Gem + ONC201**


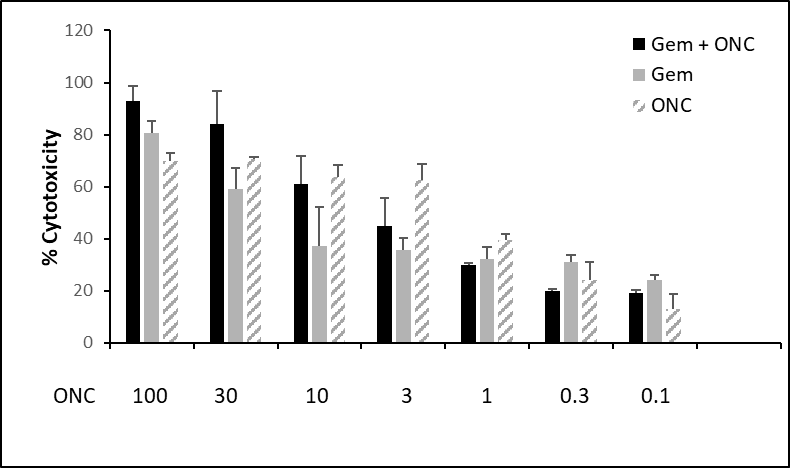


**c) SiHa – Dox + ONC201**

**
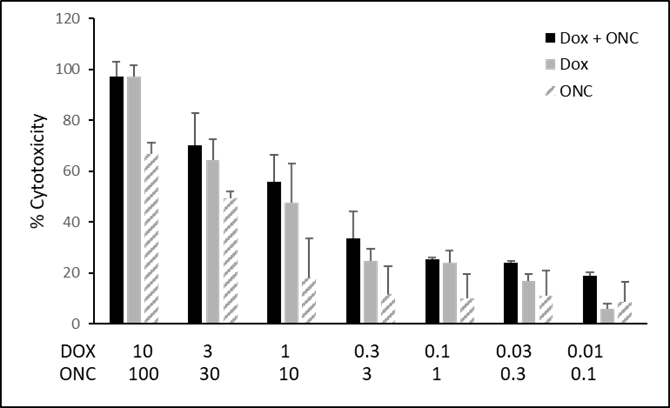
**

**d) SiHa – Gem + ONC201**


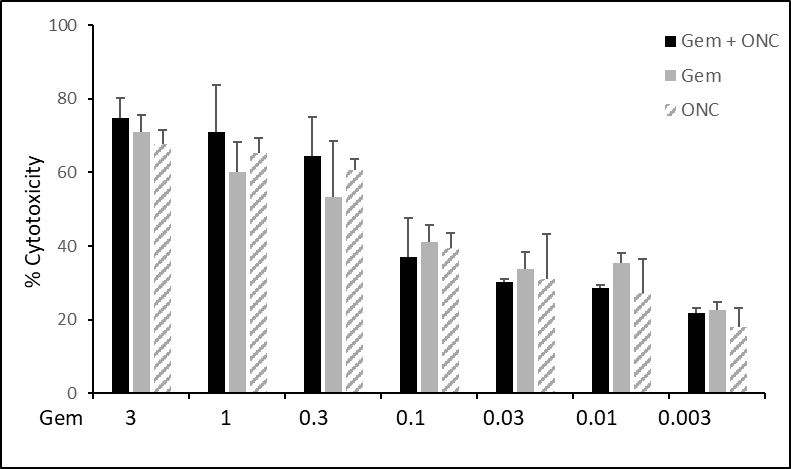

Supplement: Supplementary file 1 [file biomolecules-15-00463-s001.zip › biomolecules-3487362-supplementary new version/Figure S4_simultaneous combination bar graphs.docx]
