# Supplementary material for: ONC201 (Dordaviprone) Induces Integrated Stress Response and Death in Cervical Cancer Cells
Source: Biomolecules. 2025 Mar 21;15(4):463. doi: 10.3390/biom15040463 (PMC12025107; doi:10.3390/biom15040463)
Supplement: Supplementary file 1 [file biomolecules-15-00463-s001.zip › biomolecules-3487362-supplementary new version/Table S1- PCR primers list.docx]

**Table S1 - List of primers used for real-time PCR**

| **Gene symbol** | **Annealing temperature (°C)** | **Primer sequence (5’-3’)** | | **Product size (bp)** |
| --- | --- | --- | --- | --- |
|  |  | **Forward primer** | **Reverse primer** |  |
| TRAIL | 55 | GCTCGTTAGAAAGACTCCAAG | CTTTCCAGGTCAGTTAGCCA | 454 |
| DR5 | 55 | CTCTGAGACAGTGCTTCGAT | GTGATGTTGGATGGGAGAGT | 453 |
| BNIP3 | 60 | TGGACGGAGTAGCTCCAAGA | CGCCTTCCAATATAGATCCCCAA | 396 |
| clPp | 55 | TTGCCAGCCTTGTTATCGCA | GGTTGAGGATGTACTGCATCG | 141 |
| Beclin-1 | 51 | ACCTCAGCCGAAGACTGAAG | AACAGCGTTTGTAGTTCTGACA | 165 |
| 18s rRNA | 60 | GGAGAGGGAGCCTGAGAAAC | CCTCCAATGGATCCTCGTTA | 171 |
