# Supplementary material for: ONC201 (Dordaviprone) Induces Integrated Stress Response and Death in Cervical Cancer Cells
Source: Biomolecules. 2025 Mar 21;15(4):463. doi: 10.3390/biom15040463 (PMC12025107; doi:10.3390/biom15040463)
Supplement: Supplementary file 1 [file biomolecules-15-00463-s001.zip › biomolecules-3487362-supplementary new version/Table S2 - antibody details.docx]

**Supplementary file**

**Table S2 - Details of antibodies**

| **Name of antibody** | **company** | **Molecular weight (kDa)** | **Dilution** | **Source** | **Incubating conditions** | **Catalog number** |
| --- | --- | --- | --- | --- | --- | --- |
| Bcl-xL | Cell signaling technology | 30 | 1:1000 | Rabbit | Incubate overnight in 5% BSA, 1X TBS, 0.1% Tween 20 with gentle shaking at 4^0^C | #2764 |
| Bax | Cell signaling technology | 20 | 1:1000 | Rabbit | Incubate overnight in 5% BSA, 1X TBS, 0.1% Tween 20 with gentle shaking at 4^0^C | #2772 |
| Cyclin D1 | Cell signaling technology | 36 | 1:1000 | Rabbit | Incubate overnight in 5% BSA, 1X TBS, 0.1% Tween 20 with gentle shaking at 4^0^C | #55506 |
| ATF 4 | Cell signaling technology | 49 | 1:1000 | Rabbit | Incubate overnight in 5% BSA, 1X TBS, 0.1% Tween 20 with gentle shaking at 4^0^C | #11815 |
| Caspase 3 | Cell signaling technology | 35,17 | 1:1000 | Rabbit | Incubate overnight in 5% BSA, 1X TBS, 0.1% Tween 20 with gentle shaking at 4^0^C | #14220 |
| Caspase 8 | Cell signaling technology | 18, 43, 57 | 1:1000 | Mouse | Incubate overnight in 5% BSA, 1X TBS, 0.1% Tween 20 with gentle shaking at 4^0^C | #9746 |
| PARP | Cell signaling technology | 89,116 | 1:1000 | Rabbit | Incubate overnight in 5% NFDM, 1X TBS, 0.1% Tween 20 with gentle shaking at 4^0^C | #9542 |
| Akt (pan) | Cell signaling technology | 60 | 1:1000 | Rabbit | Incubate overnight in 5% BSA, 1X TBS, 0.1% Tween 20 with gentle shaking at 4^0^C | #4685 |
| Phospho-Akt | Cell signaling technology | 60 | 1:1000 | Rabbit | Incubate overnight in 5% BSA, 1X TBS, 0.1% Tween 20 with gentle shaking at 4^0^C | #9271 |
| Erk 1/2 | Cell signaling technology | 42,44 | 1:1000 | Rabbit | Incubate overnight in 5% BSA, 1X TBS, 0.1% Tween 20 with gentle shaking at 4^0^C | #9102 |
| Phospho Erk 1/2 | Cell signaling technology | 42,44 | 1:2000 | mouse | Incubate overnight in 5% NFDM, 1X TBS, 0.1% Tween 20 with gentle shaking at 4^0^C | #9106 |
| Bcl-2 | Cell signaling technology | 26 | 1:1000 | Rabbit | Incubate overnight in 5% NFDM, 1X TBS, 0.1% Tween 20 with gentle shaking at 4^0^C | #4223 |
| p53 | Santa cruz biotechnology | 53 | 1:500 | Mouse | Incubate overnight in 5% NFDM, 1X TBS, 0.1% Tween 20 with gentle shaking at 4^0^C | Sc-126 |
| Beta actin | Sigma | 42 | 1:5000 | Mouse | Incubate overnight in 5% NFDM, 1X TBS, 0.1% Tween 20 with gentle shaking at 4^0^C | A2228 |
| Mouse anti-rabbit IgG HRP | Santa cruz biotechnology | - | 1:1000 | Mouse | Incubate at room temperature with gentle shaking for 1.5 h | sc-2357 |
| m-IgGκ BP-HRP | Santa cruz biotechnology | - | 1:1000 | - | Incubate at room temperature with gentle shaking for 1.5 h | sc-516102 |
