# Supplementary material for: ONC201 (Dordaviprone) Induces Integrated Stress Response and Death in Cervical Cancer Cells
Source: Biomolecules. 2025 Mar 21;15(4):463. doi: 10.3390/biom15040463 (PMC12025107; doi:10.3390/biom15040463)
Supplement: Supplementary file 1 [file biomolecules-15-00463-s001.zip › biomolecules-3487362-supplementary new version/Table S3_combination.docx]

**Table S3 – Combination studies index values**

**a) Combination index values and their indication**

| **CI** | **Fa** | **Grade** | **Synergism/antagonism** |
| --- | --- | --- | --- |
| **< 0.1** | **> 0.5** | ++++ | Very highly synergistic |
| **< 0.1 - 0.4** | **> 0.5** | +++ | Highly synergistic |
| **< 0.4 - 0.8** | **> 0.5** | ++ | Moderately synergistic |
| **0.8-1.2** | **Any** | + | Additive |
| **> 1.2** | **Any** | - | Antagonism |

**b) Combination index (CI) and fraction affected (Fa) of sequential dual combinations of standard drugs (doxorubicin or gemcitabine) and ONC201 in CC cell lines**

Highly synergistic combinations are highlighted in yellow.

**i) HeLa – Dox + ONC201 (moderate to high synergism)**

| **Dox (µM)** | **ONC201 (µM)** | **Fraction affected (Fa)** | **Combination index (CI)** |
| --- | --- | --- | --- |
| 1 | 100 | 0.77 | 0.65 |
| 0.3 | 30 | 0.66 | 0.83 |
| 0.1 | 10 | 0.66 | 0.29 |
| 0.03 | 3 | 0.51 | 0.50 |
| 0.01 | 1 | 0.43 | 0.40 |
| 0.003 | 0.3 | 0.26 | 0.91 |
| 0.001 | 0.1 | 0.24 | 0.41 |

**ii) HeLa – Gem + ONC201 (moderate synergism to antagonism)**

| **Gem (µM)** | **ONC201 (µM)** | **Fraction affected (Fa)** | **Combination index (CI)** |
| --- | --- | --- | --- |
| 3 | 100 | 0.82 | 0.58 |
| 1 | 30 | 0.75 | 0.61 |
| 0.3 | 10 | 0.61 | 1.26 |
| 0.1 | 3 | 0.59 | 0.46 |
| 0.03 | 1 | 0.43 | 0.85 |
| 0.01 | 0.3 | 0.26 | 2.29 |
| 0.003 | 0.1 | 0.22 | 1.48 |

**iii) SiHa – Dox + ONC201 (moderate synergism to antagonism)**

| **Dox (µM)** | **ONC201 (µM)** | **Fraction affected (Fa)** | **Combination index (CI)** |
| --- | --- | --- | --- |
| 10 | 100 | 0.97 | 0.53 |
| 3 | 30 | 0.52 | 1.16 |
| 1 | 10 | 0.28 | 2.57 |
| 0.3 | 3 | 0.21 | 1.79 |
| 0.1 | 1 | 0.14 | 1.94 |
| 0.03 | 0.3 | 0.14 | 0.58 |
| 0.01 | 0.1 | 0.12 | 0.30 |

**iv) SiHa – Gem + ONC201 (moderate synergism to antagonism)**

| **Gem (µM)** | **ONC201 (µM)** | **Fraction affected (Fa)** | **Combination index (CI)** |
| --- | --- | --- | --- |
| 3 | 100 | 0.74 | 0.86 |
| 1 | 30 | 0.68 | 0.61 |
| 0.3 | 10 | 0.61 | 0.47 |
| 0.1 | 3 | 0.33 | 4.75 |
| 0.03 | 1 | 0.32 | 1.71 |
| 0.01 | 0.3 | 0.33 | 0.47 |
| 0.003 | 0.1 | 0.17 | 2.36 |

c) **Combination index (CI) and fraction affected (Fa) of simultaneous dual combinations of standard drugs (doxorubicin or gemcitabine) and ONC201 in CC cell lines**

**i) HeLa – Dox + ONC201**

| **Dox (µM)** | **ONC201 (µM)** | **Fraction affected (Fa)** | **Combination index (CI)** |
| --- | --- | --- | --- |
| 1 | 100 | 0.93 | 1.1 |
| 0.3 | 30 | 0.7 | 11.06 |
| 0.1 | 10 | 0.6 | 9.03 |
| 0.03 | 3 | 0.36 | 19.8 |
| 0.01 | 1 | 0.36 | 6.74 |
| 0.003 | 0.3 | 0.24 | 6.56 |
| 0.001 | 0.1 | 0.22 | 2.69 |

**ii) HeLa – Gem + ONC201**

| **Gem (µM)** | **ONC201 (µM)** | **Fraction affected (Fa)** | **Combination index (CI)** |
| --- | --- | --- | --- |
| 3 | 100 | 0.93 | 3.57 |
| 1 | 30 | 0.84 | 5.59 |
| 0.3 | 10 | 0.61 | 15.9 |
| 0.1 | 3 | 0.45 | 15.2 |
| 0.03 | 1 | 0.3 | 15.9 |
| 0.01 | 0.3 | 0.26 | 6.82 |
| 0.003 | 0.1 | 0.23 | 3.02 |

**iii) SiHa – Dox + ONC201**

| **Dox (µM)** | **ONC201 (µM)** | **Fraction affected (Fa)** | **Combination index (CI)** |
| --- | --- | --- | --- |
| 10 | 100 | 0.98 | 0.92 |
| 3 | 30 | 0.67 | 19.47 |
| 1 | 10 | 0.48 | 18.7 |
| 0.3 | 3 | 0.25 | 22.4 |
| 0.1 | 1 | 0.24 | 8.07 |
| 0.03 | 0.3 | 0.23 | 2.61 |
| 0.01 | 0.1 | 0.2 | 1.11 |

**iv) SiHa – Gem + ONC201**

| **Gem (µM)** | **ONC201 (µM)** | **Fraction affected (Fa)** | **Combination index (CI)** |
| --- | --- | --- | --- |
| 3 | 100 | 0.74 | 1.9 |
| 1 | 30 | 0.65 | 2.87 |
| 0.3 | 10 | 0.61 | 1.82 |
| 0.1 | 3 | 0.34 | 36.3 |
| 0.03 | 1 | 0.23 | 94.6 |
| 0.01 | 0.3 | 0.21 | 44.2 |
| 0.003 | 0.1 | 0.11 | 265.5 |
