# Supplementary figures and images for: ONC201 (Dordaviprone) Induces Integrated Stress Response and Death in Cervical Cancer Cells
Source: Biomolecules. 2025 Mar 21;15(4):463. doi: 10.3390/biom15040463 (PMC12025107; doi:10.3390/biom15040463)

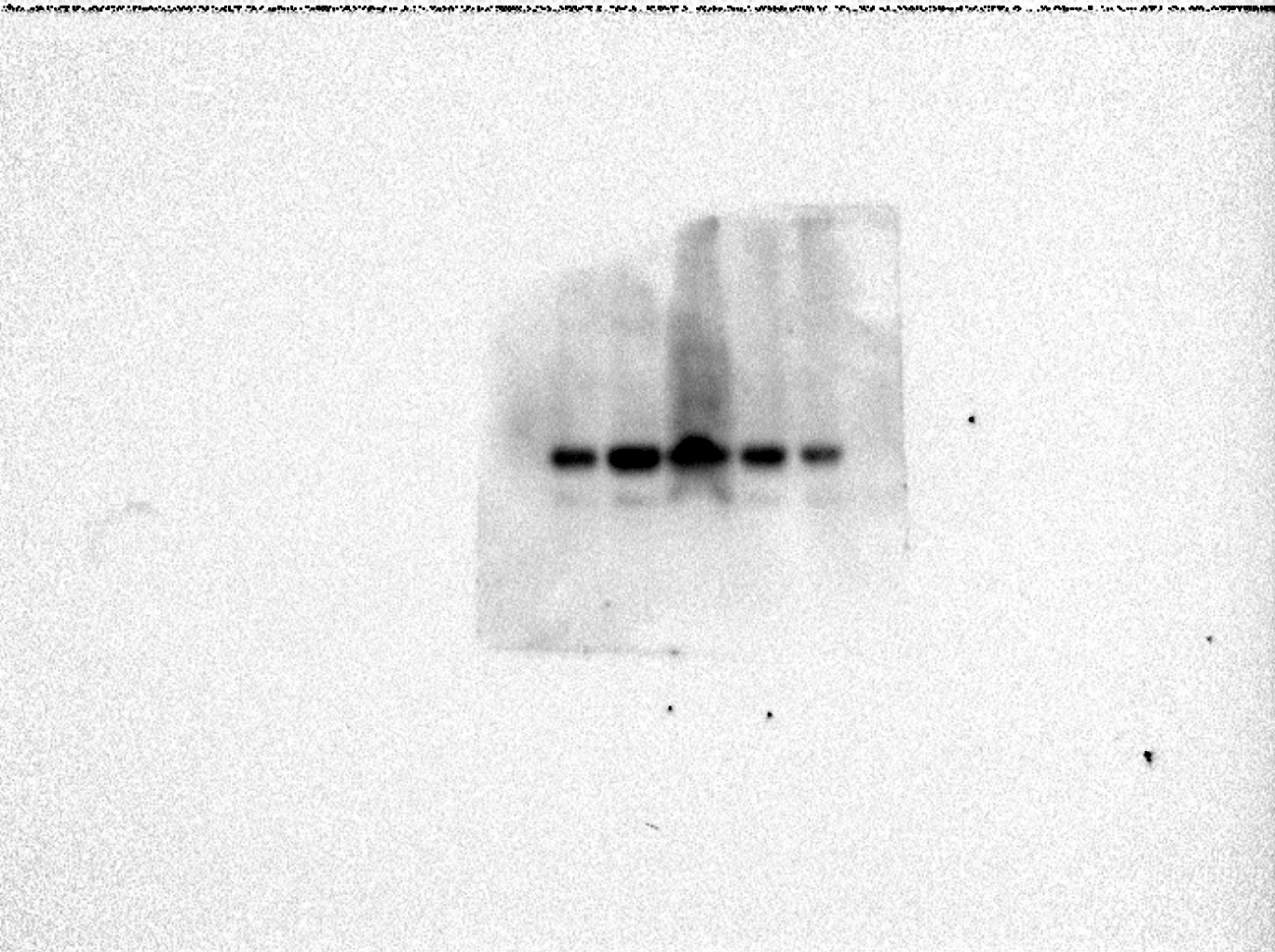

Supplement: Supplementary file 1 [file biomolecules-15-00463-s001.zip › biomolecules-3487362-supplementary new version/File S1/Hela actin_Fig 2B.jpg]

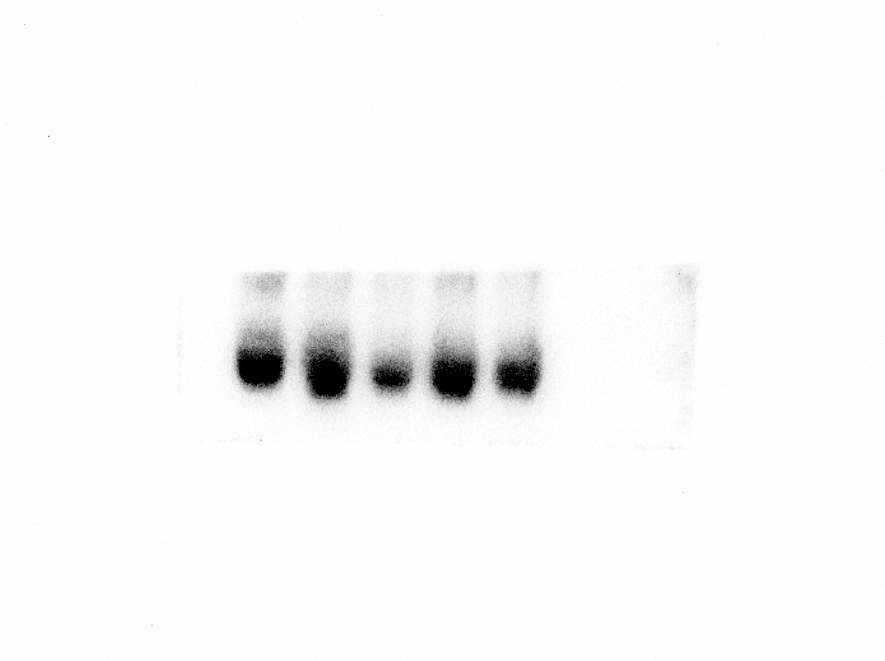

Supplement: Supplementary file 1 [file biomolecules-15-00463-s001.zip › biomolecules-3487362-supplementary new version/File S1/Hela Akt_Fig 3B.jpg]

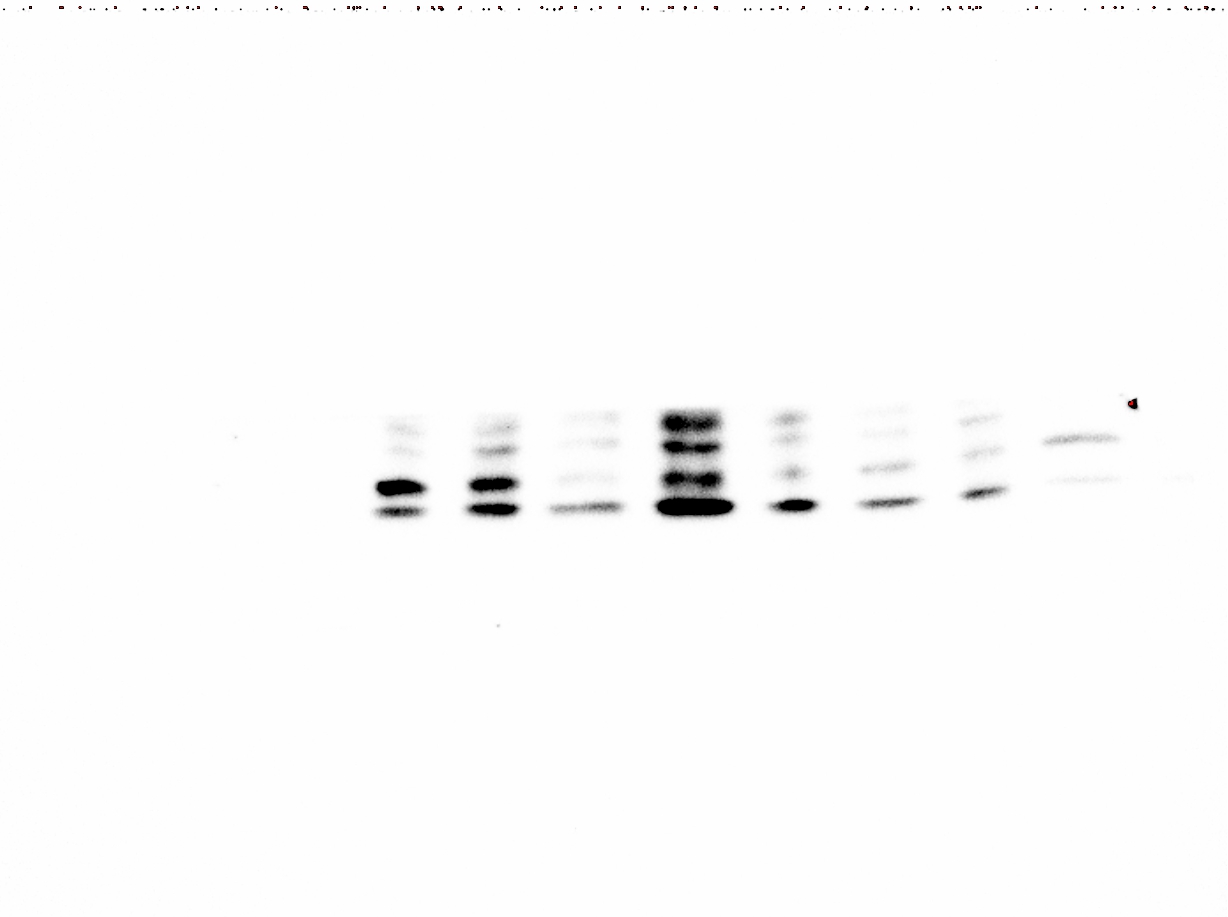

Supplement: Supplementary file 1 [file biomolecules-15-00463-s001.zip › biomolecules-3487362-supplementary new version/File S1/Hela Bax_Fig 4D.jpg]

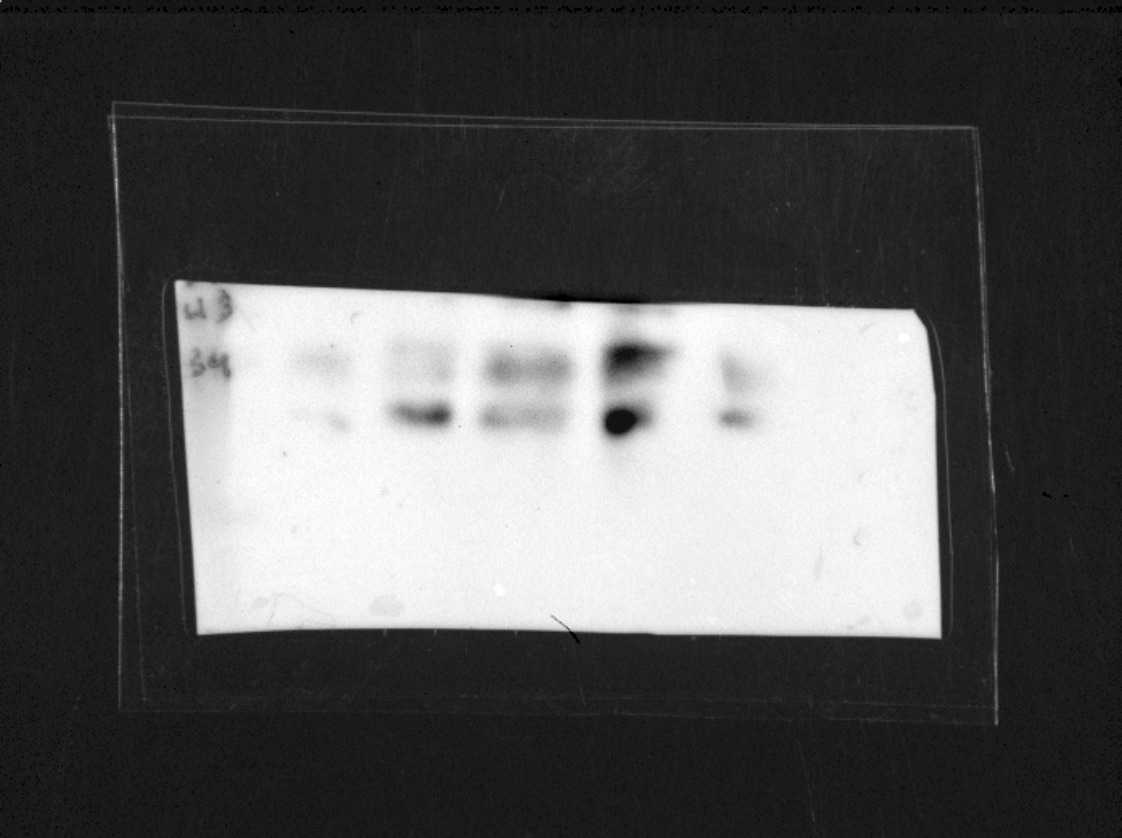

Supplement: Supplementary file 1 [file biomolecules-15-00463-s001.zip › biomolecules-3487362-supplementary new version/File S1/hela bcl2_Fig 4D.jpg]

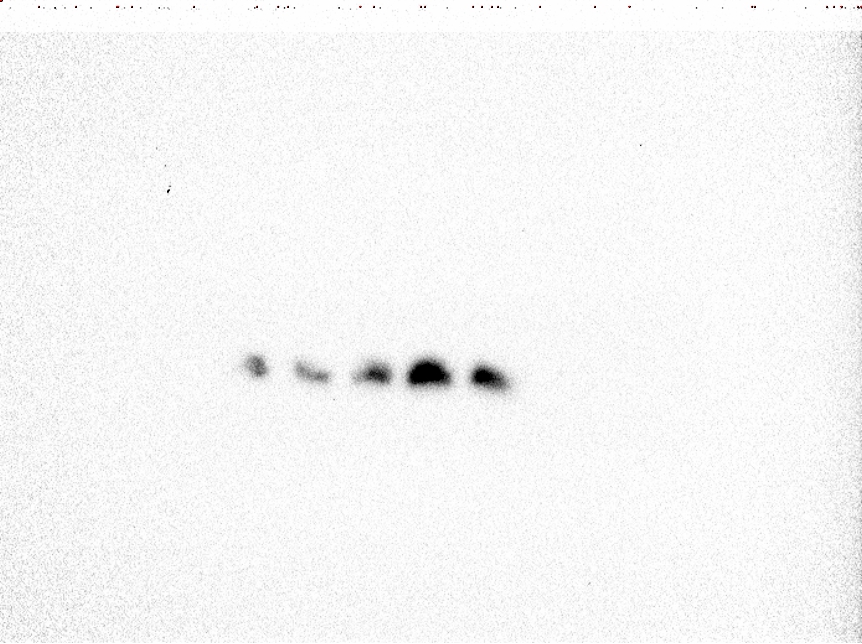

Supplement: Supplementary file 1 [file biomolecules-15-00463-s001.zip › biomolecules-3487362-supplementary new version/File S1/Hela Bclxl 48 hrs.jpg]

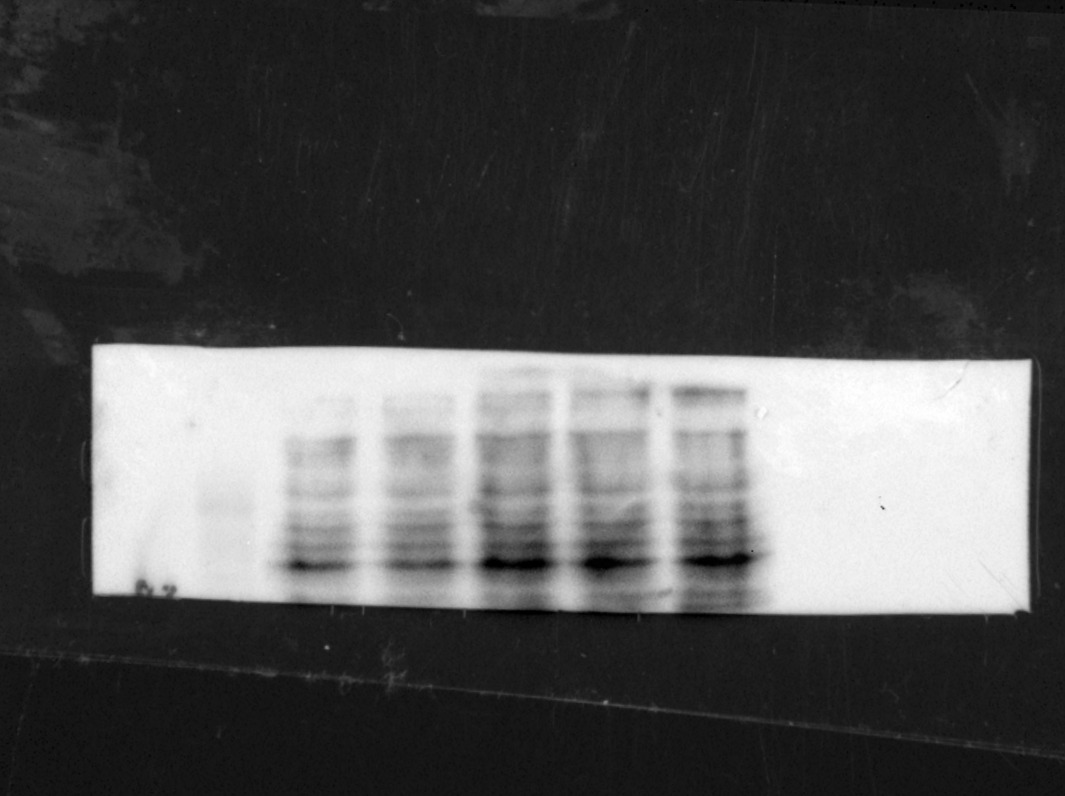

Supplement: Supplementary file 1 [file biomolecules-15-00463-s001.zip › biomolecules-3487362-supplementary new version/File S1/Hela Casp 8_Fig 4D.jpg]

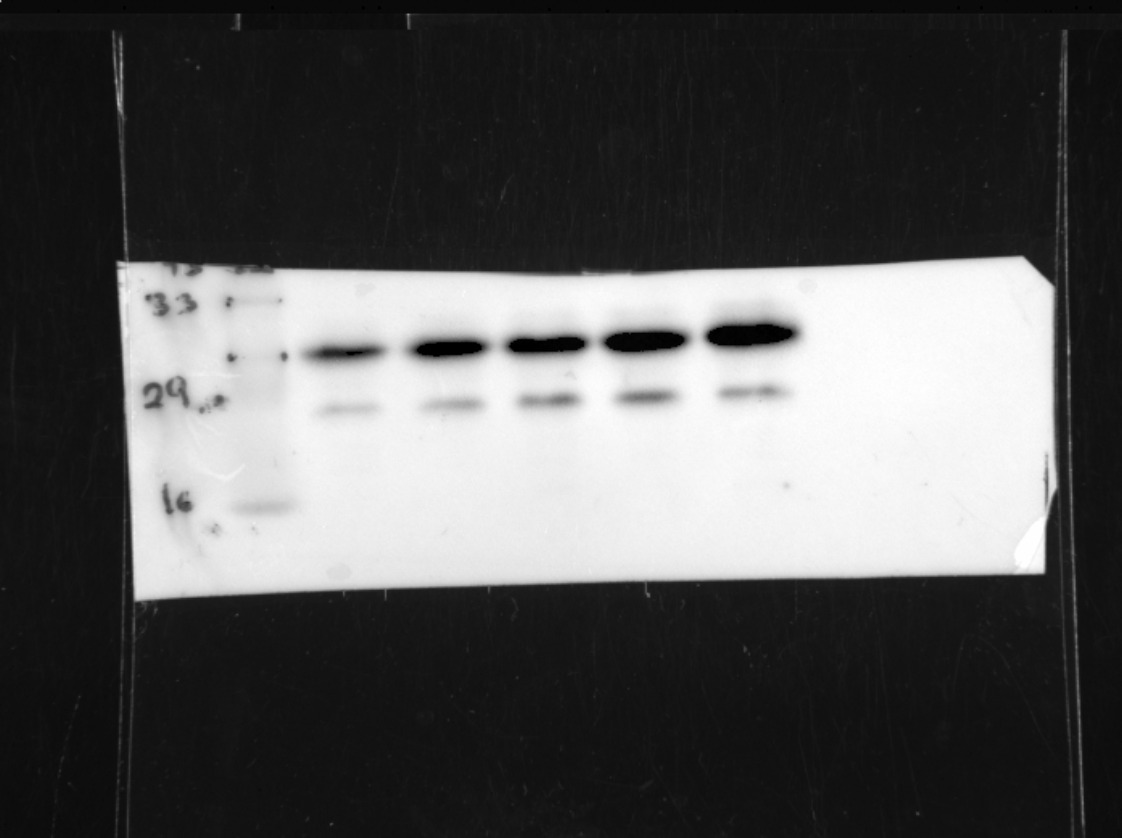

Supplement: Supplementary file 1 [file biomolecules-15-00463-s001.zip › biomolecules-3487362-supplementary new version/File S1/Hela Casp3_Fig4D.jpg]

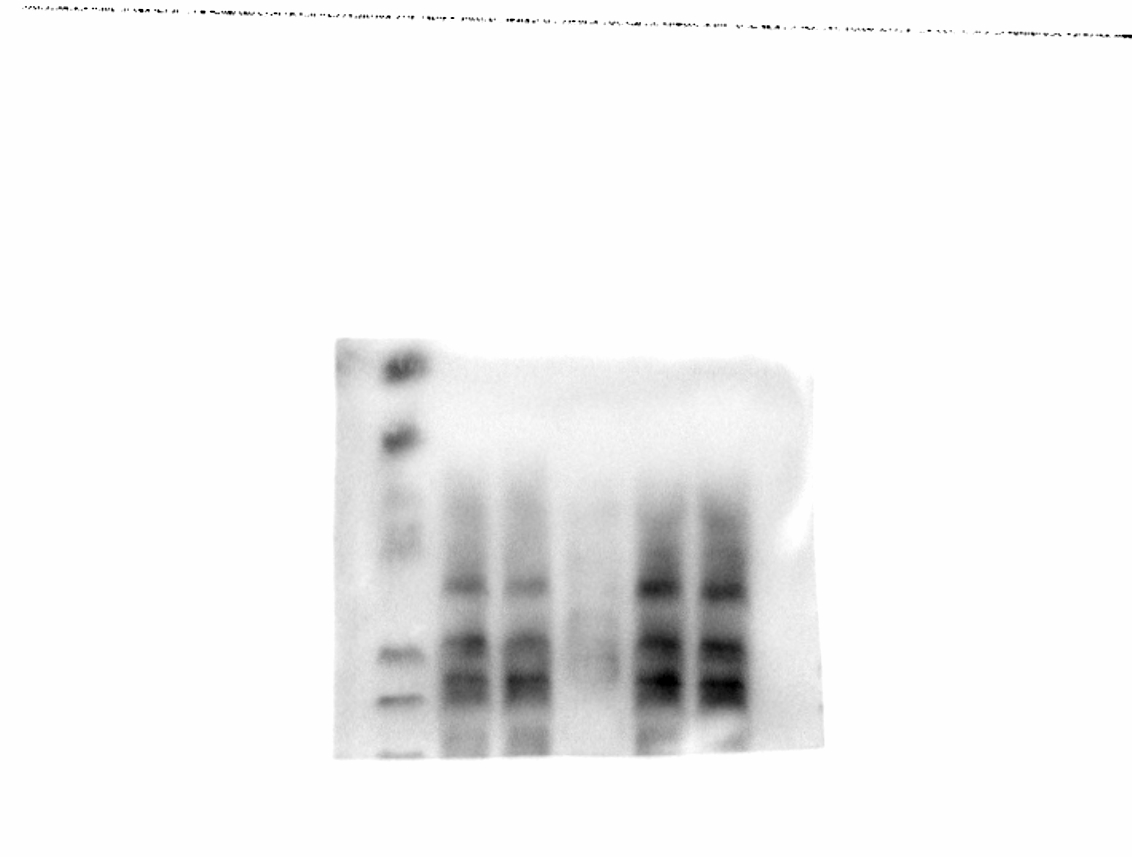

Supplement: Supplementary file 1 [file biomolecules-15-00463-s001.zip › biomolecules-3487362-supplementary new version/File S1/Hela cyclin D1_Fig 2B.jpg]

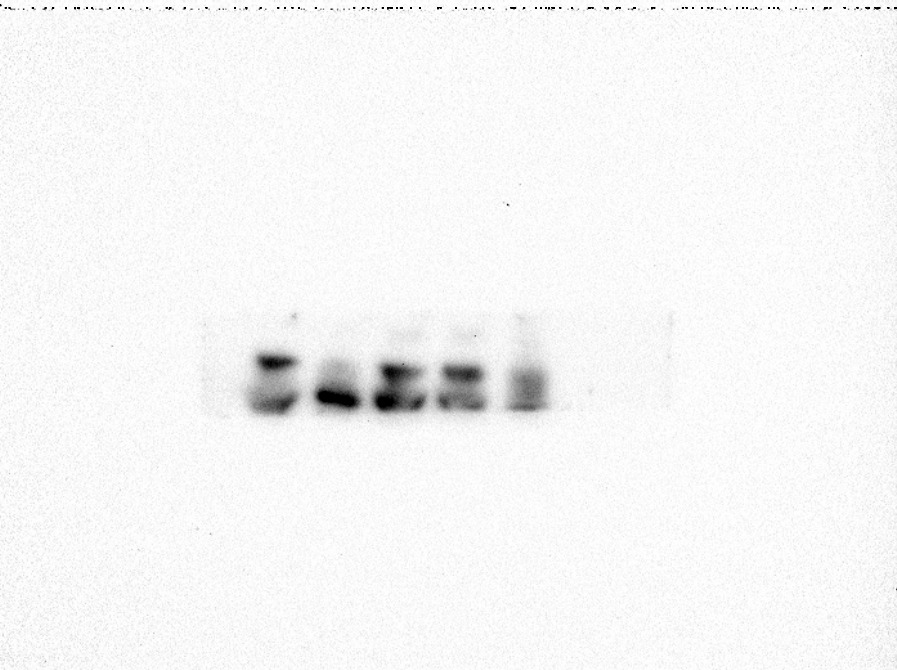

Supplement: Supplementary file 1 [file biomolecules-15-00463-s001.zip › biomolecules-3487362-supplementary new version/File S1/Hela Erk__Fig 3B.jpg]

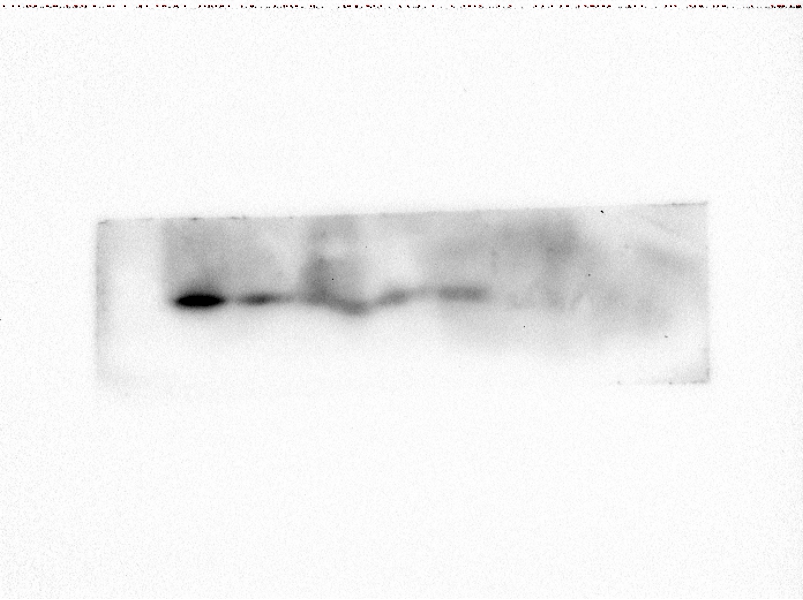

Supplement: Supplementary file 1 [file biomolecules-15-00463-s001.zip › biomolecules-3487362-supplementary new version/File S1/Hela p53_Fig 2B.jpg]

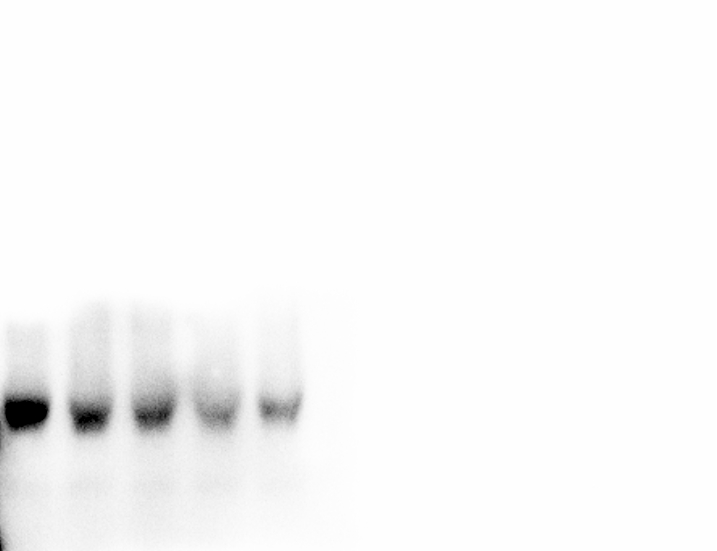

Supplement: Supplementary file 1 [file biomolecules-15-00463-s001.zip › biomolecules-3487362-supplementary new version/File S1/Hela pAkt_Fig 3B.jpg]

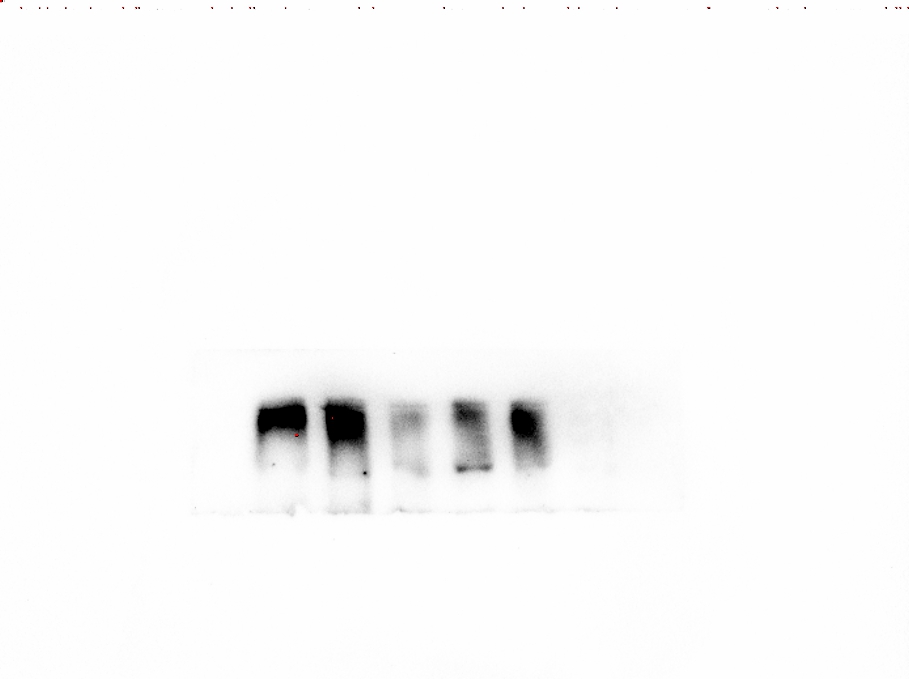

Supplement: Supplementary file 1 [file biomolecules-15-00463-s001.zip › biomolecules-3487362-supplementary new version/File S1/Hela PARP_Fig 4D.jpg]

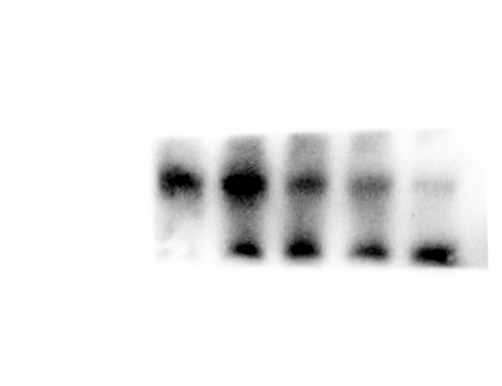

Supplement: Supplementary file 1 [file biomolecules-15-00463-s001.zip › biomolecules-3487362-supplementary new version/File S1/Hela pErk_Fig 3B.jpg]

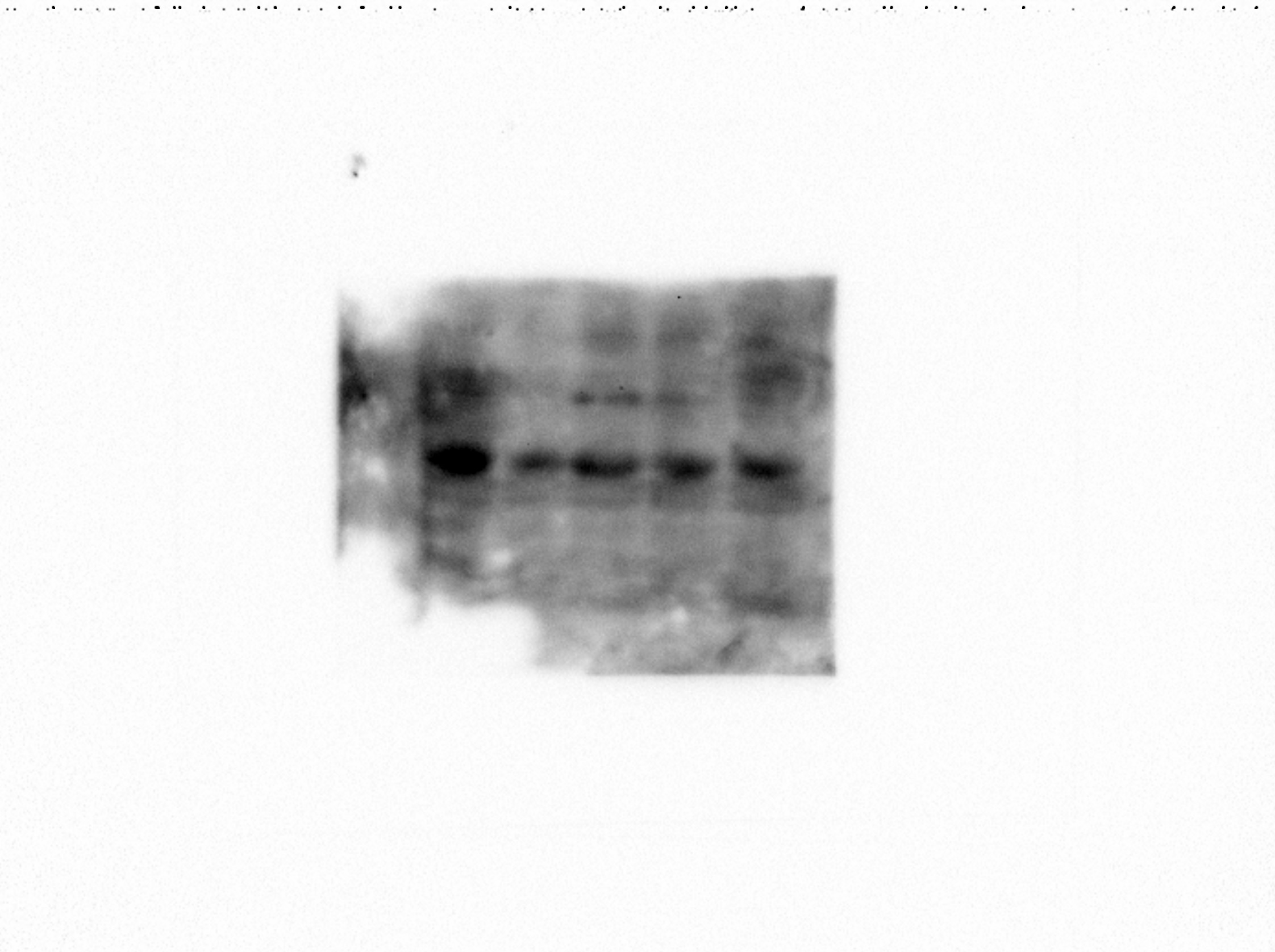

Supplement: Supplementary file 1 [file biomolecules-15-00463-s001.zip › biomolecules-3487362-supplementary new version/File S1/Hela_actin_Fig 3A.jpg]

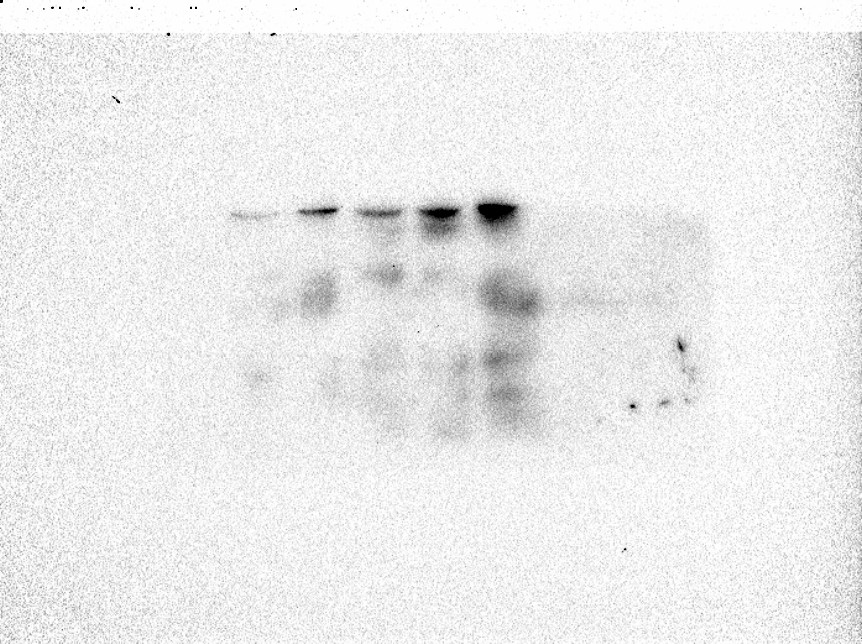

Supplement: Supplementary file 1 [file biomolecules-15-00463-s001.zip › biomolecules-3487362-supplementary new version/File S1/Hela_ATF4_Fig3A.jpg]

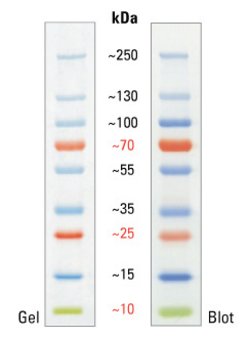

Supplement: Supplementary file 1 [file biomolecules-15-00463-s001.zip › biomolecules-3487362-supplementary new version/File S1/Pageruler_prestained ladder_thermo_26619.jpg]

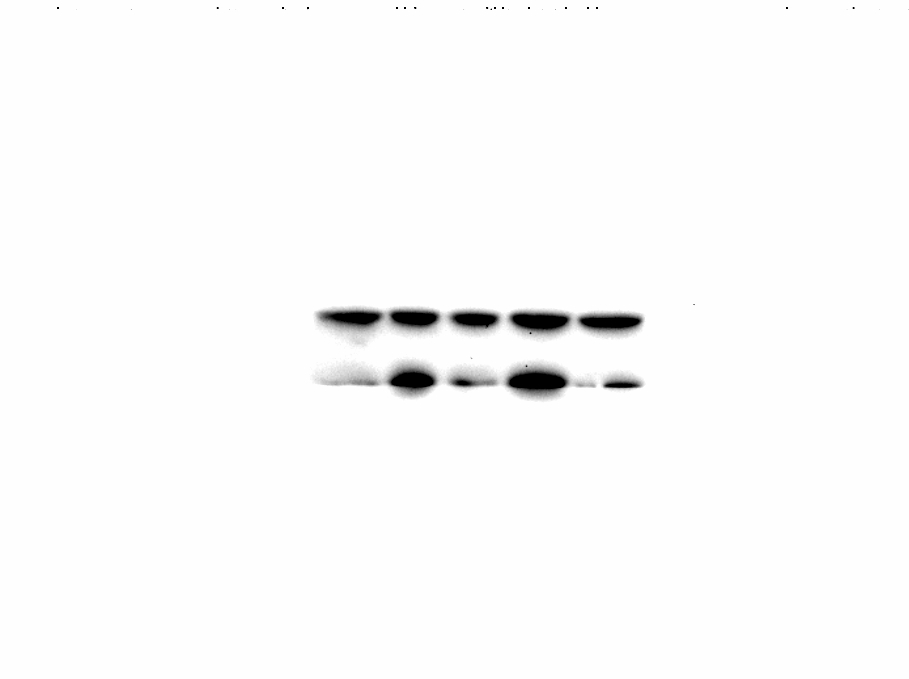

Supplement: Supplementary file 1 [file biomolecules-15-00463-s001.zip › biomolecules-3487362-supplementary new version/File S1/SIha actin Fig 3A.jpg]

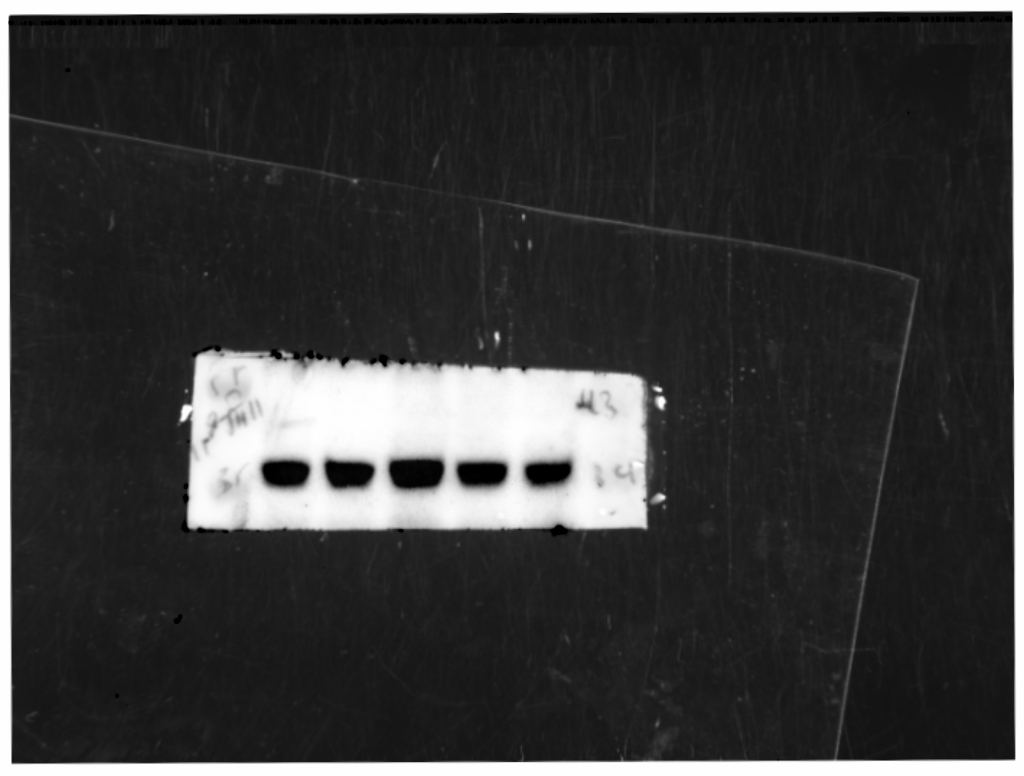

Supplement: Supplementary file 1 [file biomolecules-15-00463-s001.zip › biomolecules-3487362-supplementary new version/File S1/Siha actin_Fig 2B.jpg]

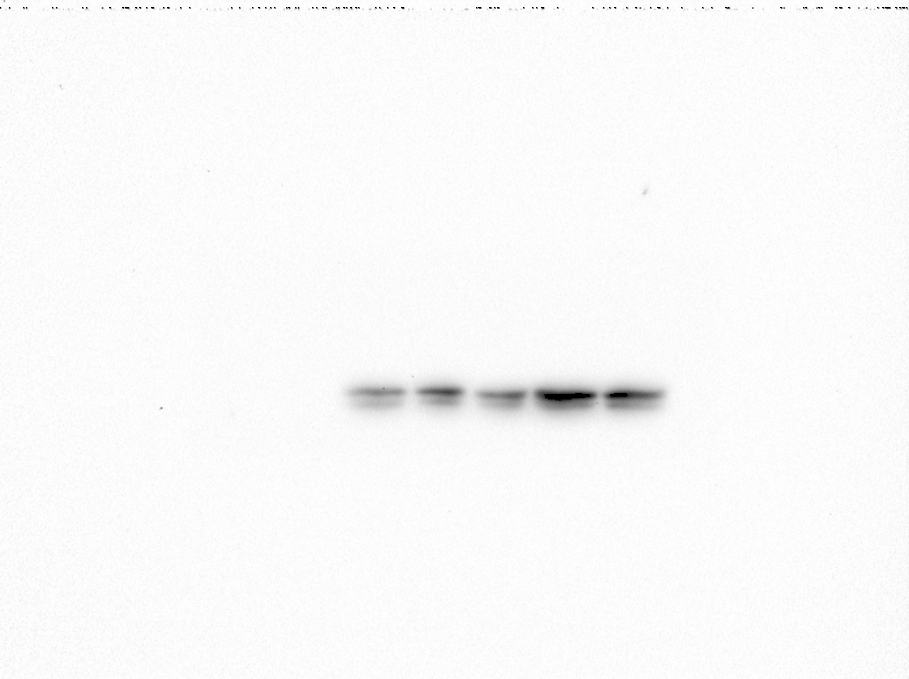

Supplement: Supplementary file 1 [file biomolecules-15-00463-s001.zip › biomolecules-3487362-supplementary new version/File S1/Siha Akt_Fig 3B.jpg]

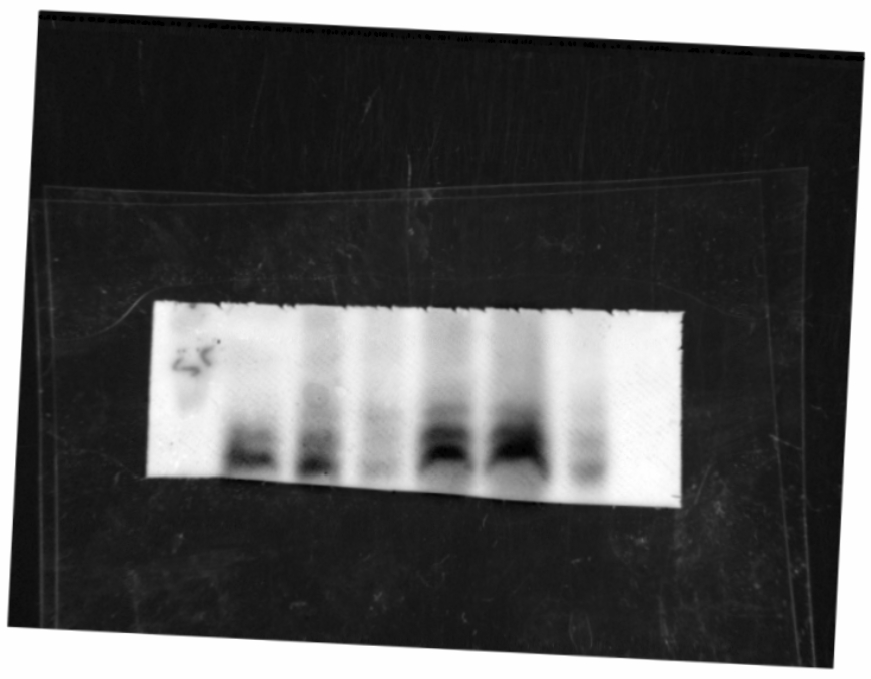

Supplement: Supplementary file 1 [file biomolecules-15-00463-s001.zip › biomolecules-3487362-supplementary new version/File S1/Siha ATF4 Fig3A.jpg]

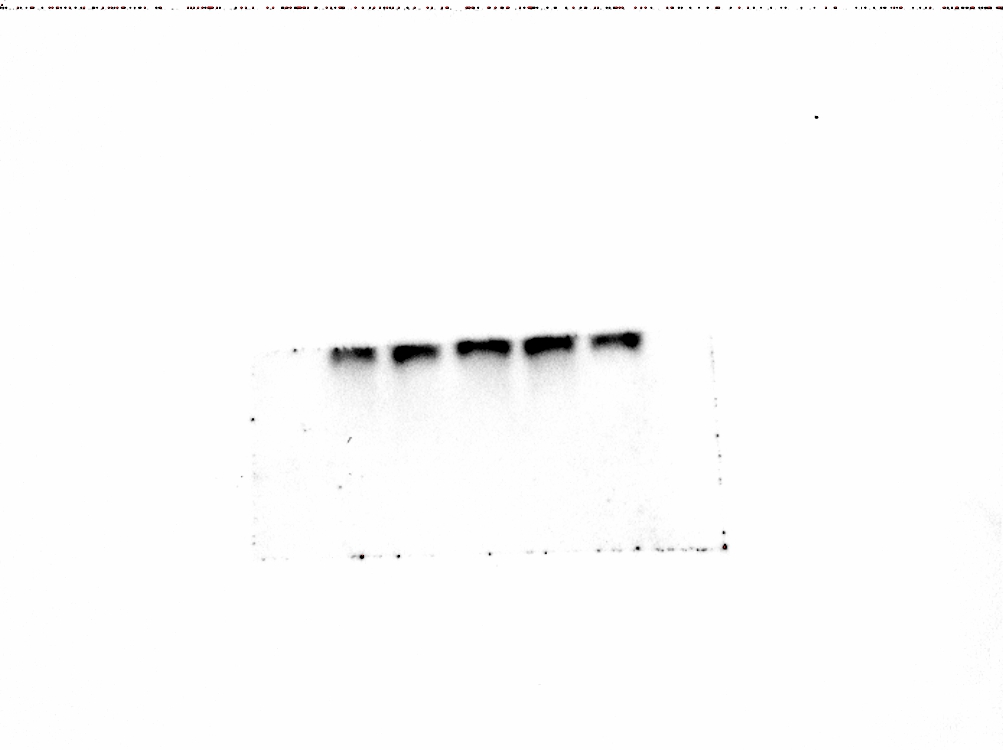

Supplement: Supplementary file 1 [file biomolecules-15-00463-s001.zip › biomolecules-3487362-supplementary new version/File S1/Siha Bax_Fig 4D.jpg]

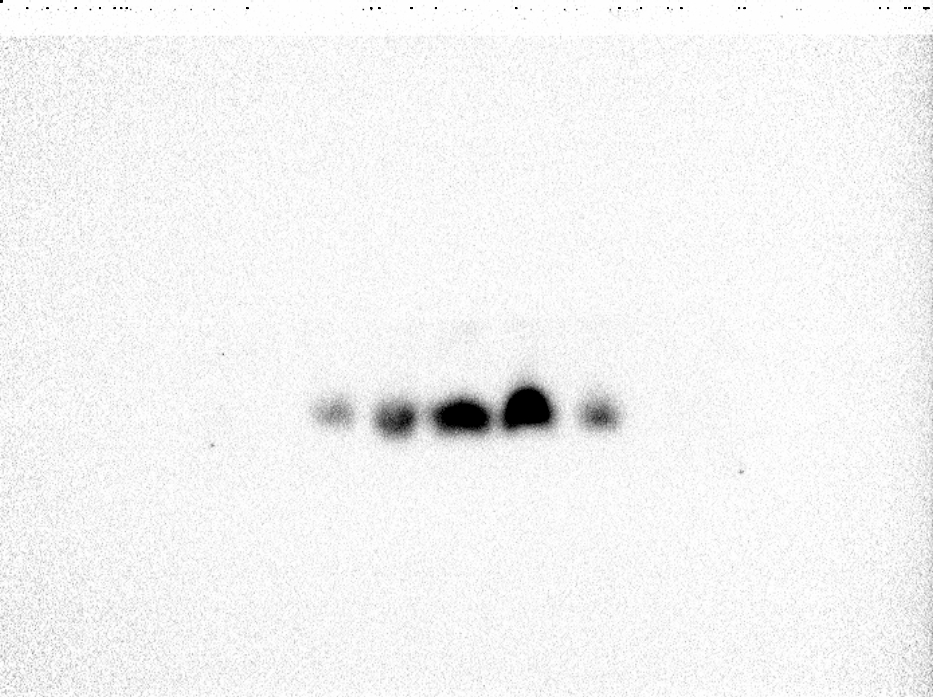

Supplement: Supplementary file 1 [file biomolecules-15-00463-s001.zip › biomolecules-3487362-supplementary new version/File S1/Siha Bclxl_Fig4D.jpg]

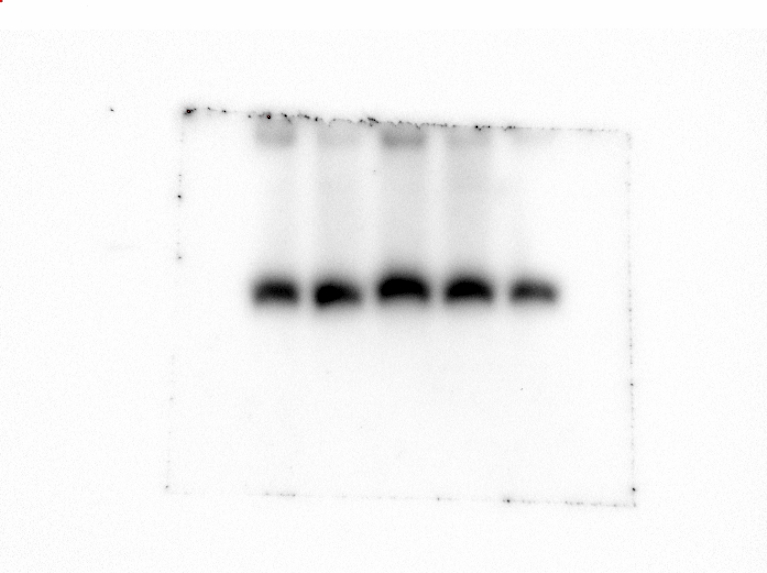

Supplement: Supplementary file 1 [file biomolecules-15-00463-s001.zip › biomolecules-3487362-supplementary new version/File S1/Siha Casp3_Fig 4D.jpg]

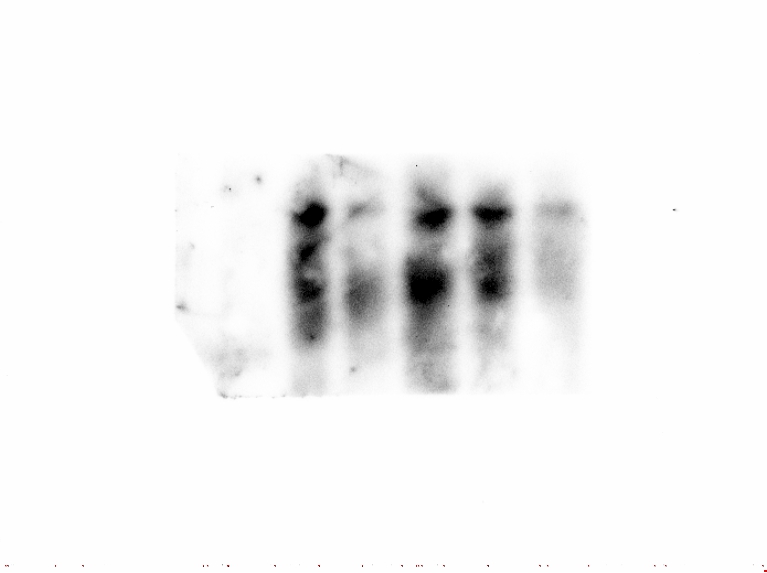

Supplement: Supplementary file 1 [file biomolecules-15-00463-s001.zip › biomolecules-3487362-supplementary new version/File S1/Siha cyclin D1_Fig 2B.jpg]

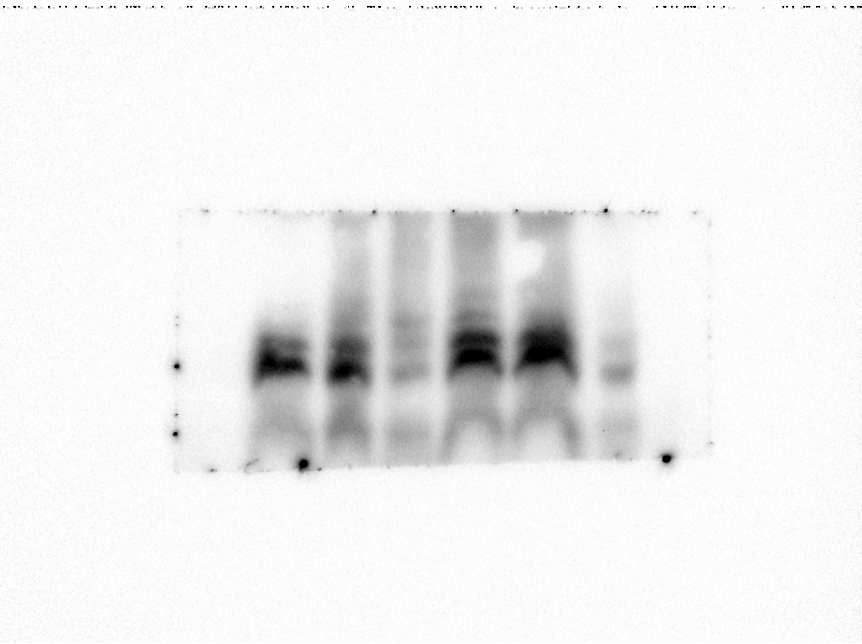

Supplement: Supplementary file 1 [file biomolecules-15-00463-s001.zip › biomolecules-3487362-supplementary new version/File S1/Siha Erk_Fig3B.jpg]

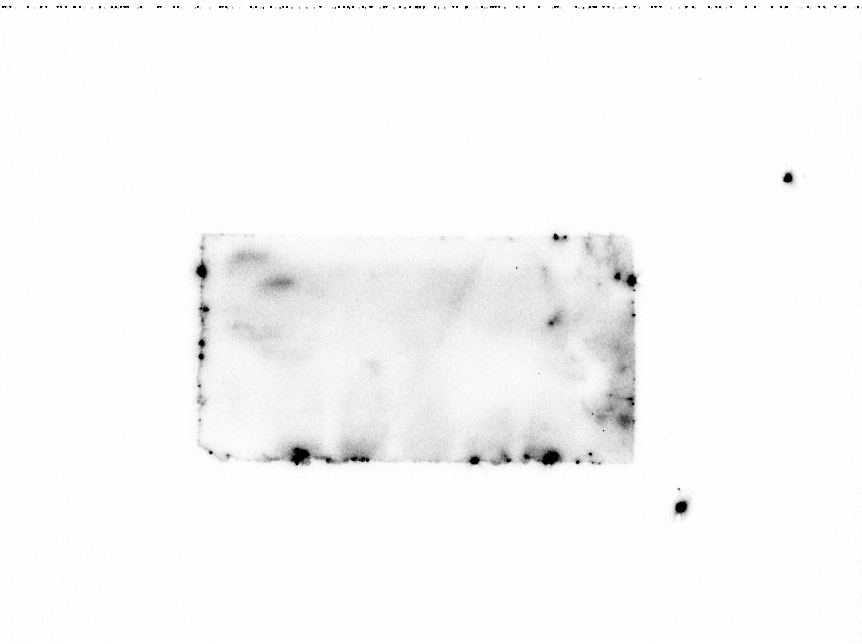

Supplement: Supplementary file 1 [file biomolecules-15-00463-s001.zip › biomolecules-3487362-supplementary new version/File S1/Siha p53_Fig 2B.jpg]

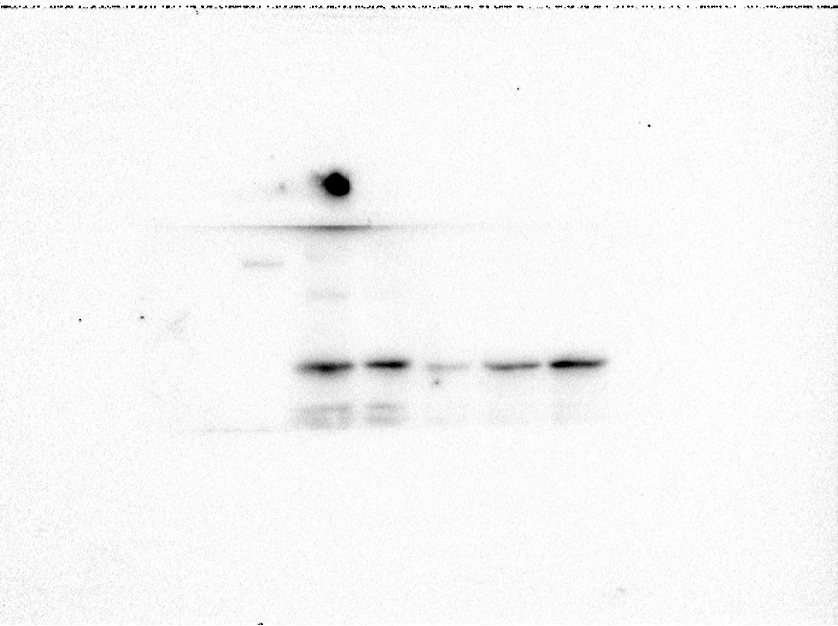

Supplement: Supplementary file 1 [file biomolecules-15-00463-s001.zip › biomolecules-3487362-supplementary new version/File S1/Siha pAkt_Fig 3B.jpg]

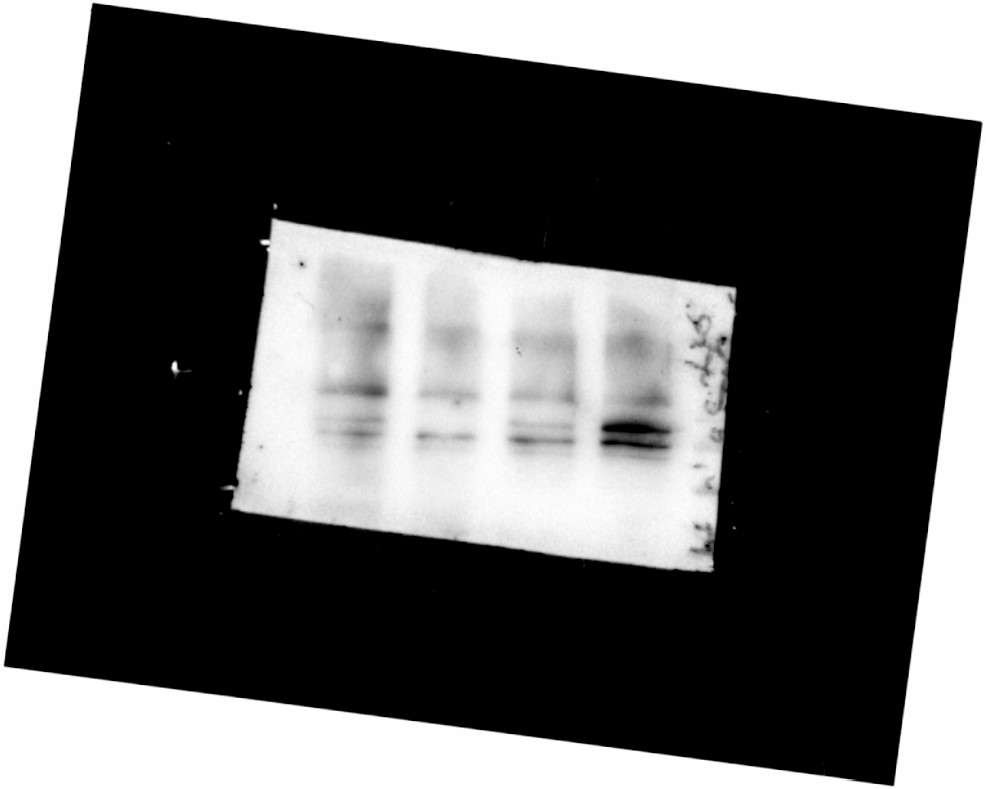

Supplement: Supplementary file 1 [file biomolecules-15-00463-s001.zip › biomolecules-3487362-supplementary new version/File S1/Siha PARP_Fig 4D.jpg]

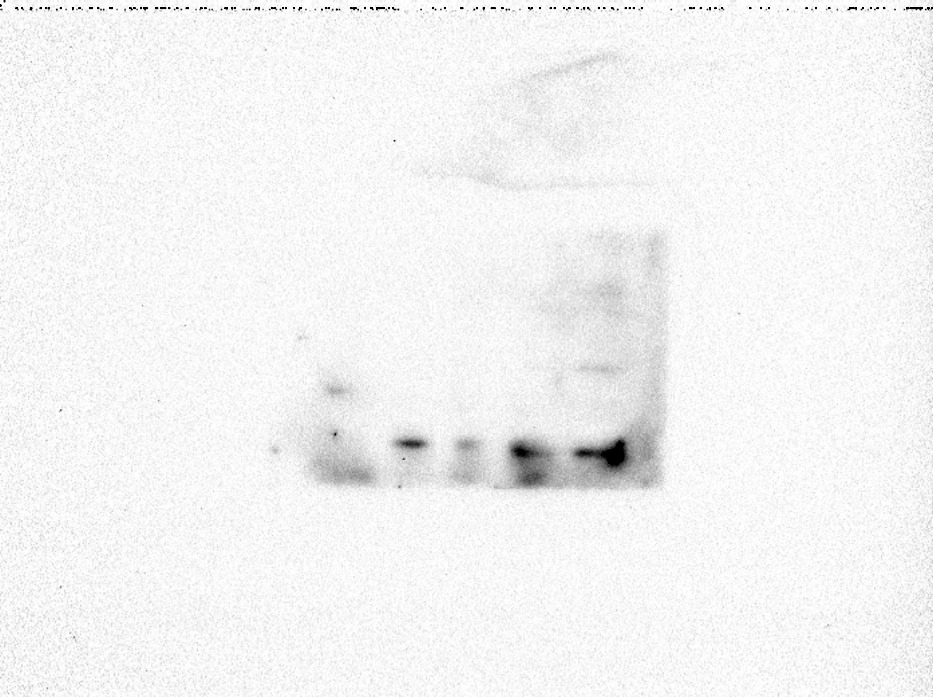

Supplement: Supplementary file 1 [file biomolecules-15-00463-s001.zip › biomolecules-3487362-supplementary new version/File S1/Siha_pErk_Fig3B.jpg]
